# White matter fiber bundles enable robust conduction and time-critical signaling despite axon caliber variation

**DOI:** 10.1038/s42003-026-11206-8

**Published:** 2026-10-27

**Authors:** Laurin Mordhorst, Nikolaus Weiskopf, Markus Morawski, Siawoosh Mohammadi

**Affiliations:** 1 Department of Neuroradiology, University of Luebeck, Luebeck, Germany; 2 Institute of Systems Neuroscience, University Medical Center Hamburg-Eppendorf, Hamburg, Germany; 3 Department of Neurophysics, Max Planck Institute for Human Cognitive and Brain Sciences, Leipzig, Germany; 4 Felix Bloch Institute for Solid State Physics, Faculty of Physics and Earth System Sciences, Leipzig University, Leipzig, Germany; 5 Functional Imaging Laboratory, Department of Imaging Neuroscience, UCL Queen Square Institute of Neurology, University College London, London, UK; 6 Paul Flechsig Institute - Centre of Brain Research and Neuropathology, Leipzig, Germany; 7 Max Planck Research Group MRI Physics, Max Planck Institute for Human Development, Berlin, Germany

**Keywords:** Neural circuits, Action potential generation, Biophysical models

## Abstract

Axon caliber determines signal conduction velocity and varies within and across the bundles that form the brain’s wiring. Our knowledge of this organization derives largely from 2D histology, assuming cylindrical axons whose calibers are described by their radius. However, recent 3D histology reveals substantial along-axon variability in radius, calling into question 2D-derived insights into axon radius and conduction velocity. We show in 450,000 3D axon reconstructions from male rats that—despite this individual variation—axon bundles possess stable radius distributions at the ensemble level. These distributions are faithfully captured by 2D cross-sections and yield robust conduction velocity predictions, as along-axon variability has only a modest effect. Conduction in large axons is particularly stable, supporting time-critical signaling. With 2D sampling validated, we leverage 46 million human corpus callosum axons from 2D histology to determine sample size requirements across neuroscience applications. Our findings show how axonal organization gives rise to stable ensemble statistics and conduction, explaining and reinforcing decades of 2D histology-based research.

## Introduction

The brain’s white matter connects distant regions via tracts—bundles of aligned myelinated axons. The caliber of these axons varies both within and across tracts, and is a key determinant of conduction velocity: thicker axons conduct faster, enabling rapid long-range communication^1^, while thinner axons are more numerous and metabolically efficient, serving less time-critical communication^2^. Beyond basic neuroscience, axon caliber holds promise as biomarkers for neurological disorders^3,4^ and is in principle accessible non-invasively via magnetic resonance imaging (MRI)^5–8^.

Much of our understanding of axon organization derives from 2D histological cross-sections, interpreted under the cylinder model—the assumption that axons are uniform tubes characterized by their radius^9–13^. This approach has yielded a rich body of findings. Axon radius distributions are right-skewed, with a large bulk of many thin axons and a sparse tail of few thick, fast-conducting axons—a shape often captured by parametric distributions such as the gamma or generalized extreme value distribution^5,14–16^. While the bulk of the distribution is similar across species, the length of the distribution tail varies, with larger brains extending the tail to include giant axons, partially accounting for longer conduction distances^12,17^. Within brains, tracts differ systematically: motor-related pathways contain larger axons than association tracts^10,18^, and long-range axons are thicker than short-range axons^1^. Additionally, 2D histology-derived distributions have shown consistency with functional and biophysical measurements grounded in the cylinder model, including conduction velocity measurements from invasive electrophysiology^19,20^ and human electroencephalography^16,21^, as well as axon radius measurements with diffusion MRI^7,8^.

Yet, the consistency of these findings may be surprising given known limitations of 2D histology. First, most studies use microscopy sections much narrower than the tracts they sample, with sample sizes potentially insufficient to characterize distribution tails or validate parametric radius distribution models. Second, 3D axon reconstructions from serial-section electron microscopy have revealed that individual axons deviate from the cylindrical model, exhibiting radius variation and undulations along their trajectory^15,22–26^. Though there are hints that axon radius distributions remain stable along tracts based on few (~ 50) large axons^22^, this remains to be established quantitatively at scale. Beyond histological sampling, deviations from cylindrical geometry affect biophysical modeling with diffusion MRI^15,22,27–31^ and conduction velocity predictions^32^. For conduction velocity, it is of particular interest whether axons of different sizes are differentially affected, given their distinct functional roles^2,33^. Under what conditions, then, do 2D histology-derived axon radius distributions yield meaningful functional and biophysical predictions?

Here, we study along-axon radius variation and its implications, the robustness of axon radius distributions to histological sampling (2D vs 3D, sample size), and their parametric description. Leveraging the largest available dataset of 3D rat axon reconstructions (450,000 corpus callosum and cingulum axons), we first show that radius variation along axons has modest impact on conduction velocity, with large axons enabling particularly stable conduction. We then mimic 2D histological sampling and find that 2D-derived radius distributions faithfully represent 3D bundle properties despite radius variation along individual axons. With 2D histological sampling validated, we turn to the largest available 2D human dataset (46 million corpus callosum axons) to provide sample size guidance: even small samples (~ 10^3^ axons) reasonably capture the distribution bulk, whereas larger samples (~ 10^5^ axons) are needed to capture the long distribution tail in humans. Finally, we show that this long tail challenges parametric modeling of axon radius distributions.

## Results

**Fig. 1:**
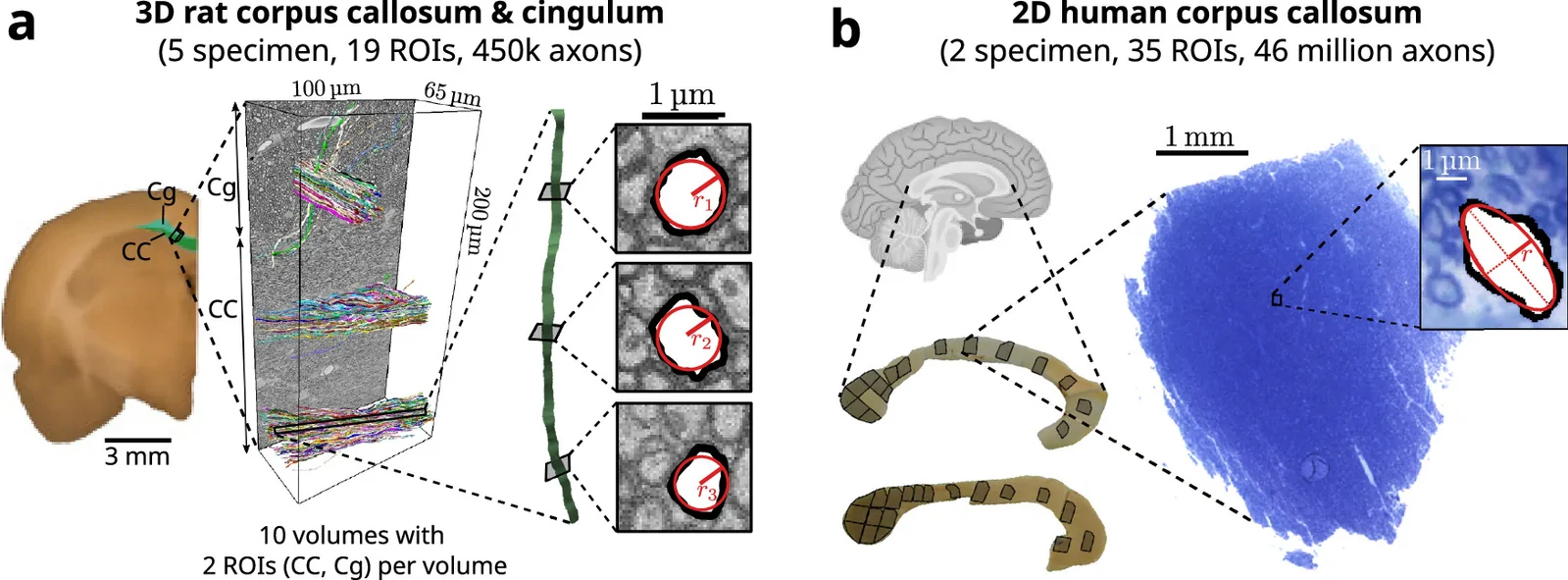
Dataset overview. **a** 3D electron microscopy dataset from rat white matter. Left: coronal view of rat brain hemisphere showing the location of the electron microscopy volume spanning corpus callosum (CC) and cingulum (Cg). Center: representative electron microscopy volume, showing the full electron microscopy section in the background and a few extracted axon bundles. The bundle orientations indicate the two tracts: most Cg axons run approximately toward the reader, most CC axons from left to right. Right: a single reconstructed axon with cross-sections at three positions along its trajectory, taken perpendicular to the centerline. In each cross-section, the radius (*r*_1_ to *r*_3_) is that of a circle with the same area as the segmented cross-section. The dataset comprises 19 ROIs distributed over 10 volumes from 5 rats (2 sham-operated controls, 3 after traumatic brain injury), totaling approximately 450,000 reconstructed axons. **b** 2D light microscopy dataset from the human corpus callosum. Left: anatomical schematic showing ROI locations across two post-mortem samples. Right: representative light microscopy image with inset showing a single segmented axon. The fitted ellipse is shown in red with its major and minor axes indicated; in contrast to 3D, the radius *r* is estimated from the minor axis, which is more robust to obliquely cut axons than the circular equivalent radius. The dataset comprises 35 ROIs totaling 46 million segmented axons. Rat brain schematic and electron microscopy volume in (**a**) were adapted from ref. ^35^, licensed under CC BY 4.0.

We extracted axon radii from two complementary deep white matter datasets (see Fig. 1): 3D electron microscopy of rat corpus callosum and cingulum (~ 450,000 reconstructed axons across 19 ROIs, see Fig. 1a), and 2D light microscopy of human corpus callosum (~ 46 million axons across 35 ROIs, see Fig. 1b). The 3D data enables analysis along individual axon trajectories; the 2D data provides extensive sampling across distinct axons.

### Along-axon radius variation has modest impact on conduction velocity

**Fig. 2:**
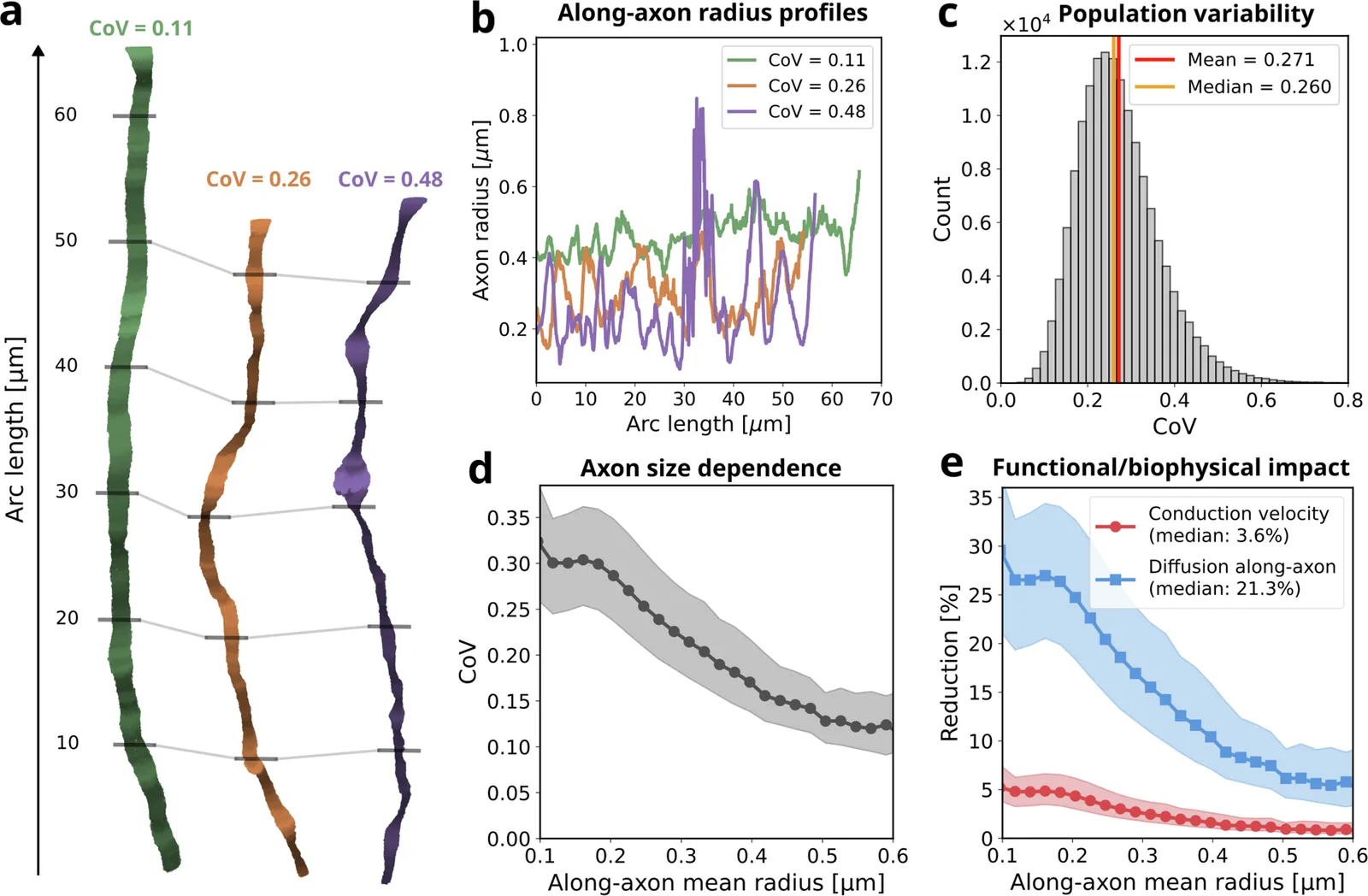
Along-axon radius variation and its impact. **a** 3D renderings of three representative rat corpus callosum axons with different levels of along-axon radius variation. Variation is quantified by the coefficient of variation (CoV; see Eq. (1)), annotated above each axon. Brightness indicates local radius (dark = low, bright = high). **b** Radius profiles along arc length for the axons in (**a**), with radius measured perpendicular to the centerline. **c** Distribution of CoV across all axons; vertical lines indicate mean and median. **d** CoV as a function of along-axon mean radius. Axons are binned by along-axon mean radius; line shows median, shaded region shows interquartile range (IQR). **e** Estimated reduction of conduction velocity (see Eq. (4)) and along-axon diffusion (see Eq. (7)) due to radius variation; 0% corresponds to ideal cylindrical axons without radius variation (CoV = 0). Data are displayed as in (**d**). The legend provides the median reduction across all axons. Statistic computation in (**c**–**e**) was restricted to axons exceeding 20 μm arc length (*n* ≈ 150,000) to robustly quantify along-axon CoV; bins in (**d**, **e**) were constrained to a minimum of 50 axons.

To characterize radius variation along axons, we extracted radius profiles from the 3D axon reconstructions in rats. Fig. 2a shows 3D renderings of three representative axons with low, medium, and high along-axon radius variation; Fig. 2b shows the corresponding radius along the axonal trajectories. The radius changes gradually along axons, with the thickest axon (green) showing less variation than thinner ones (orange, purple).

Moving from representative axons to population level, Fig. 2c shows the histogram of along-axon radius variation as coefficient of variation (CoV), i.e., the standard deviation of the radius relative to the along-axon mean (see Eq. (1)). The CoV has a median of 0.27, but can reach up to 0.6 for some axons. Interestingly, CoV shows a clear dependency on the mean along-axon radius (see Fig. 2d), decreasing for thicker axons. Supplementary Fig. 1 reveals the mechanism behind this: the absolute radius variation saturates for thick axons.

To assess the functional and biophysical impact of radius variation (see Fig. 2e), we estimated the CoV-related reduction of conduction velocity and along-axon diffusion—accessible in vivo with non-invasive diffusion MRI—using Eq. (4) and (7)? Conduction velocity is much less affected than along-axon diffusion, with median reductions of ~ 4 and ~ 21%, respectively. This also holds for an empirically measured, radius-dependent myelin thickness (see Supplementary Fig. 2).

### 2D cross-sections capture axon radius distributions

**Fig. 3:**
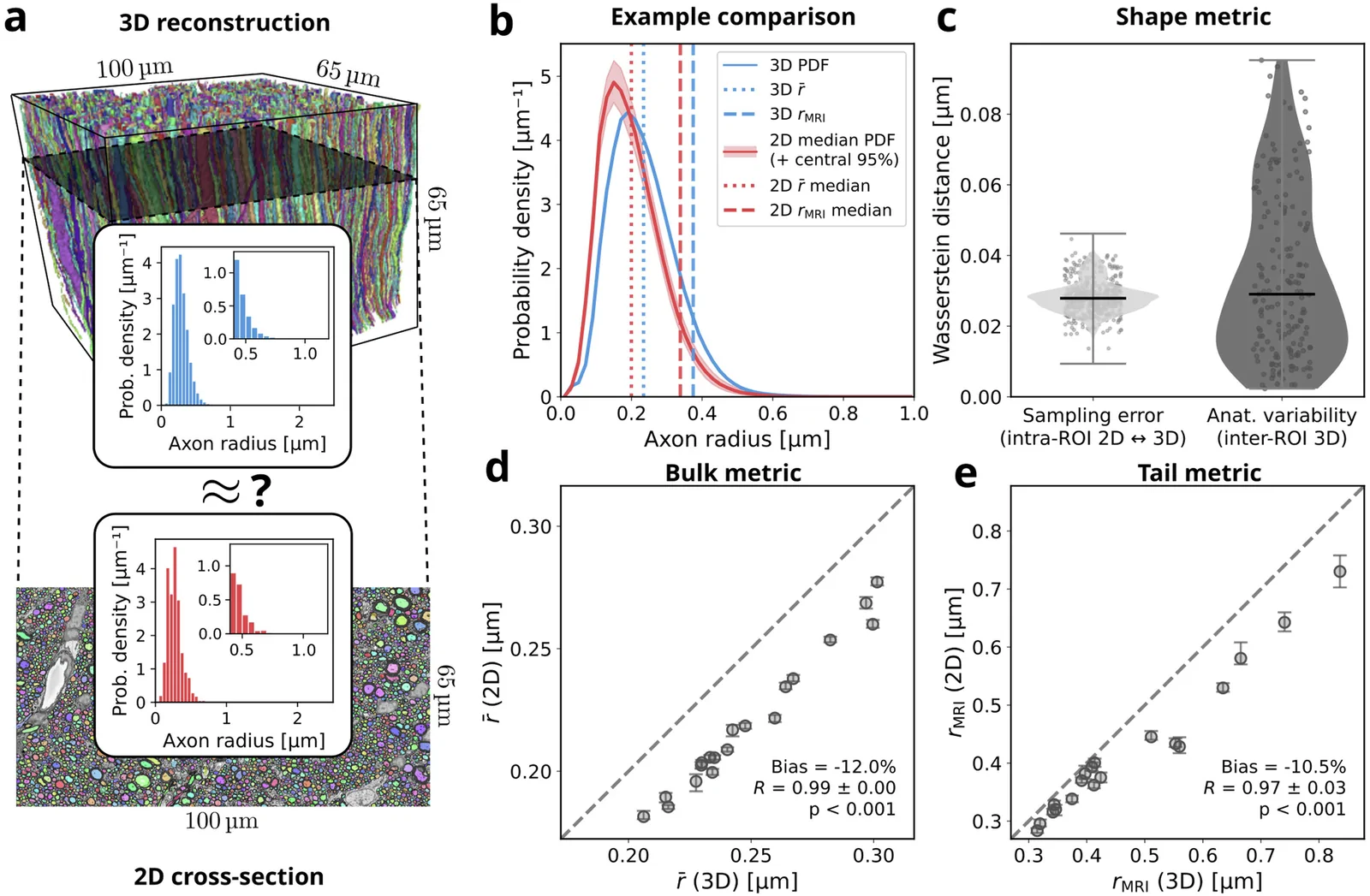
2D-derived axon radius distributions as 3D proxies. **a** 3D reconstruction of cingulum axons with corresponding 2D cross-section perpendicular to the bundle orientation, as typical for 2D histology. Insets show the corresponding axon radius distributions: 3D pools radii jointly across and along axons, whereas 2D captures only a single snapshot across axons. Do 2D distributions and derived ensemble metrics represent their 3D counterparts? **b** Example comparison for one ROI. Solid lines show 2D and 3D-based radius distributions; vertical lines mark ensemble average radii: the arithmetic mean $\bar{r}$ (dotted, see Eq. (9)) is sensitive to the distribution bulk; the MRI-visible axon radius *r*_MRI_ (dashed, see Eq. (10)) is sensitive to the distribution tail. For 2D, lines indicate median values across cross-sections; the shaded band spans the central 95% of cross-sections. **c** Distribution shape disagreement, quantified by Wasserstein distance (see Eq. (8)). The left distribution encodes all comparisons between distributions from 2D and their 3D counterpart, whereas the right distribution contextualizes this with inter-ROI distances between 3D distributions, reflecting the anatomical variation in the dataset. **d**, **e** Comparison of $\bar{r}$ and *r*_MRI_ from 2D cross-sections versus 3D reference across all ROIs. Markers show median and IQR across 2D cross-sections for each ROI; dashed lines indicate identity. Pearson’s *R* and *p*-value were computed via Monte Carlo sampling.

While individual axon radii vary along their trajectory (see Fig. 2), the axon ensemble’s radius distribution along the bundle may nonetheless remain stable. We evaluated this hypothesis by tracing local axon radius distributions along bundles, using cross-sections mimicking 2D histological sampling, and compared them to the full 3D distribution, which pools radii along and across all axons (see Fig. 3a).

Figure 3b compares the axon radius distribution from 2D cross-sections against the 3D counterpart for one representative ROI. The distributions are similar in shape, but the 2D distribution is shifted toward smaller radii, with a slightly larger bulk of small axons. To summarize the distributions, we computed two ensemble-average radius metrics sensitive to different parts of the distribution: the arithmetic mean radius $\bar{r}$ (see Eq. (9)), which reflects the distribution bulk, and *r*_MRI_ (see Eq. (10)), a radius metric that diffusion MRI is sensitive to, which is determined by the distribution tail. In correspondence with the shift between 2D and 3D distributions, both metrics are slightly lower in 2D than in 3D.

To quantify the 2D–3D correspondence across all ROIs, we assessed distribution shape via Wasserstein distance (see Eq. (8), Fig. 3c). The Wasserstein distance shows a consistent offset of approximately 0.04 μm, confirming the systematic bias of 2D cross-sections toward smaller radii. However, the spread between 2D and 3D-based distributions within each ROI is low, indicating that 2D-derived distributions are highly stable along the bundle. Moreover, this spread is much smaller than the Wasserstein distance differences due to anatomical variation across ROIs, suggesting that 2D cross-sections are sensitive to anatomical differences despite the bias.

This sensitivity to anatomical differences is confirmed by both ensemble average radii $\bar{r}$ and *r*_MRI_ (see Fig. 3d, e): 2D and 3D estimates are strongly correlated across ROIs (*R* ≥ 0.97, *p* < 0.001), despite 2D values being systematically lower (12% for $\bar{r}$, 10.5% for *r*_MRI_).

The bias between distributions and metrics (see Fig. 3b–e) likely arises from the minor axis approximation in 2D versus perpendicular-to-centerline measurements in 3D, which can be mitigated by using circular equivalent radius, albeit at the cost of lower precision (see Supplementary Fig. 3). Thus, the observed differences between 2D and 3D are driven by geometric approximations rather than a fundamental limitation of 2D sampling.

**Fig. 4:**
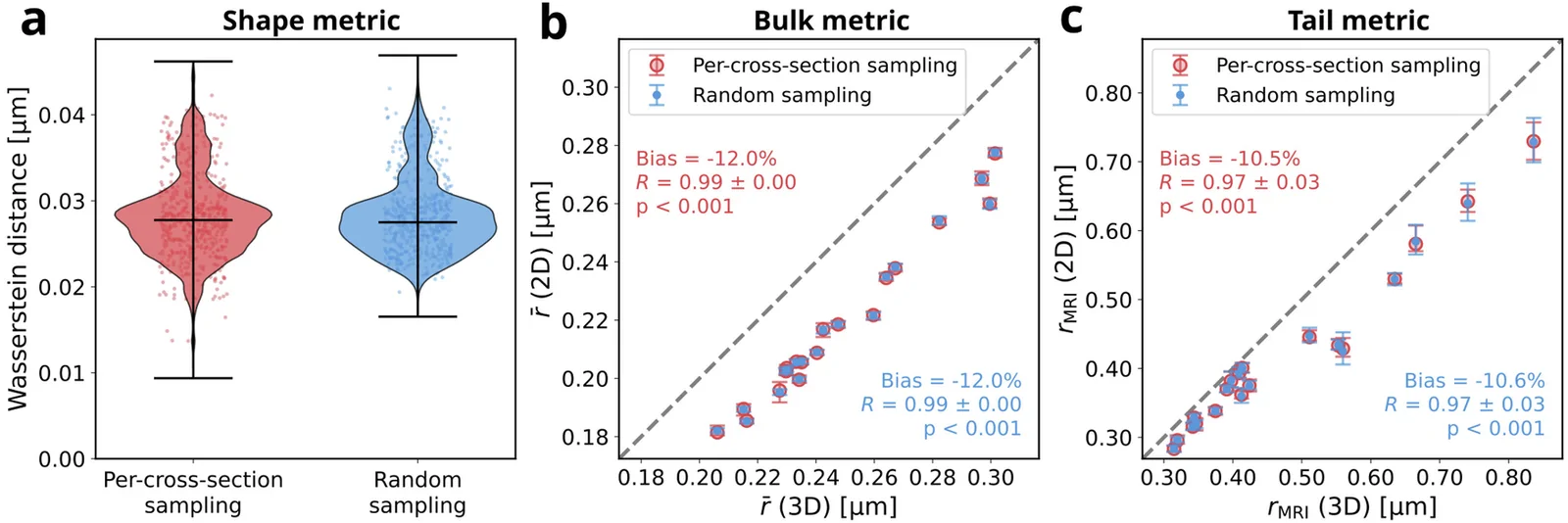
Per-cross-section versus random sampling of 2D axon radii. Monte Carlo comparison (10,000 iterations) of 2D cross-sections against random samples of matched size from the 2D radii pooled across all cross-sections of the ROI (see Fig. 3a for an example cross-section). Both are compared against the pooled 3D reference distribution for: **a** Wasserstein distance between sampled distribution and the 3D reference. Violin plots show distribution across all ROI-iteration pairs. **b** Mean radius $\bar{r}$ from 2D samples versus 3D reference. Points show median per ROI; dashed lines indicate identity. Compared to Fig. 3d, e, error bars here show IQR across Monte Carlo iterations, not across all 2D cross-sections of one ROI. **c** MRI-visible radius *r*_MRI_, format as in (**b**). Pearson’s *R* and *p*-value were computed via Monte Carlo sampling.

To test directly whether the residual 2D–3D differences reflect spatial structure within cross-sections, we compared actual cross-sections against random samples of matched size drawn from the 2D radii pooled across all cross-sections of each ROI (see Fig. 4). If axons of similar radius clustered within a cross-section, per-cross-section sampling would deviate more from the 3D reference than random sampling. Fig. 4a–c repeat the comparisons of Fig. 3c–e for both sampling schemes, covering distribution shape and the ensemble average radii $\bar{r}$ and *r*_MRI_. Both schemes agree closely, with median Wasserstein distances of 0.028 μm (per cross-section) versus 0.027 μm (random), and near-identical correlations with the 3D reference for $\bar{r}$ (*R* = 0.99 ± 0.00, *p* < 0.001 for both) and *r*_MRI_ (*R* = 0.97 ± 0.03, *p* < 0.001 for both), suggesting that axon radius is spatially unstructured within cross-sections.

### Sample size requirements differ for bulk and tail metrics

Having established that 2D versus 3D sampling does not substantially change the observed axon radius distribution, sample size remains as an important consideration. To study sample size requirements across species, we conducted a subsampling analysis using massive axon radius distributions as population reference: the 3D rat data from previous sections (> 10^6^ radii per ROI, pooled across and along axons) and 2D human corpus callosum light microscopy (~ 10^6^ axons per ROI).

**Fig. 5:**
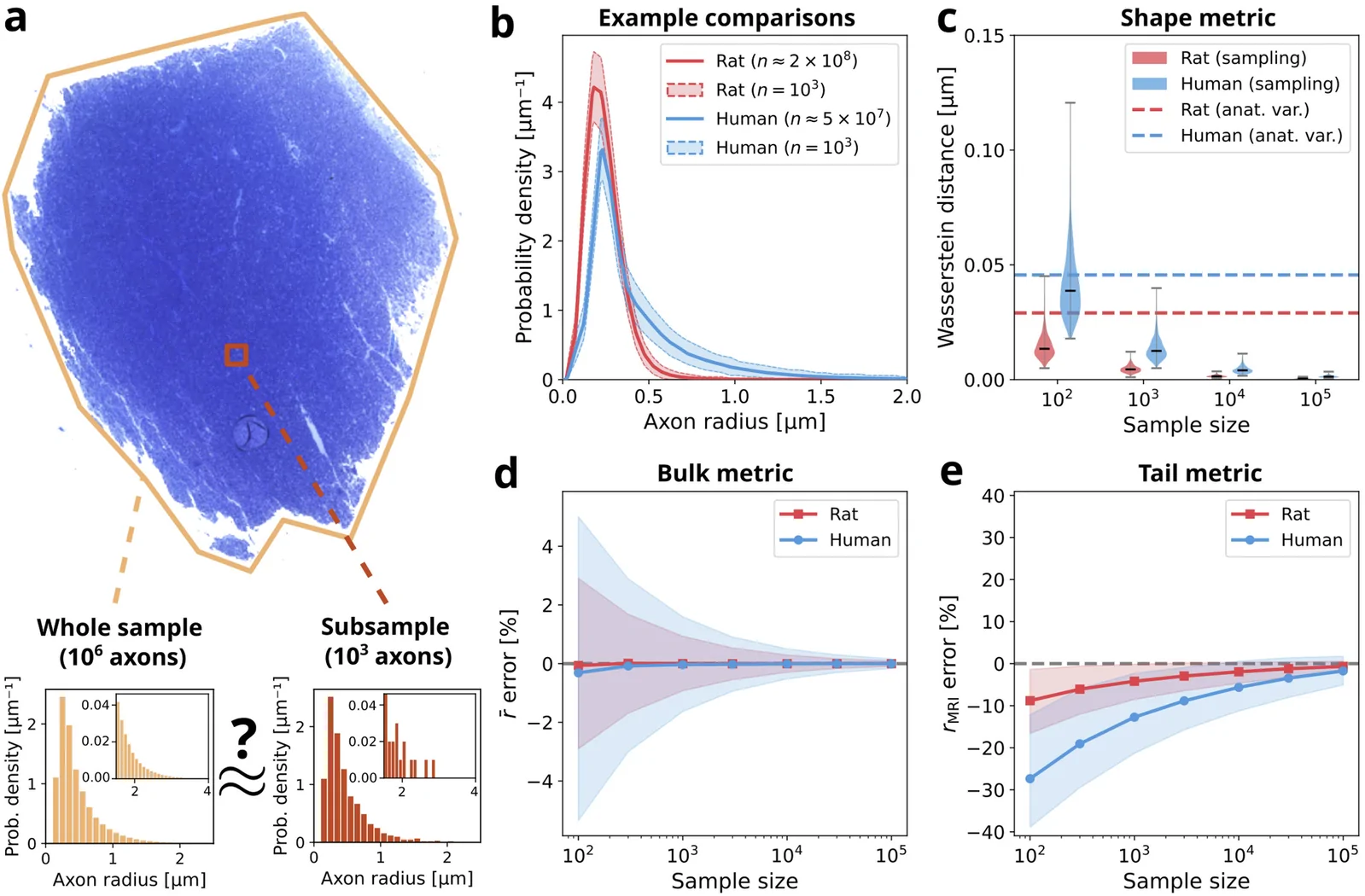
Sample size requirements for axon radius distributions. **a** Human corpus callosum light microscopy section illustrating whole sample (~ 10^6^ axons) versus subsample (10^3^ axons). Histograms show probability density for both samples; insets emphasize the tail region. **b** Probability density pooled across all ROIs for human (blue; 35 ROIs, ~ 5 × 10^7^ axons) and rat (red; 19 ROIs, ~ 2 × 10^8^ radius samples). Solid lines show full data; shaded bands show the central 95% across subsamples at *n* = 10^3^. **c** Wasserstein distance (see Eq. (8)) as a function of sample size; horizontal dashed lines indicate median inter-ROI anatomical variation for reference. **d**, **e** Relative error of ensemble average axon radii as a function of sample size: **d** arithmetic mean radius $\bar{r}$ (see Eq. (9)); **e** MRI-visible axon radius *r*_MRI_ (see Eq. (10)). Markers show median; shaded regions indicate IQR (pooled across ROIs and subsamples). All simulations in (**b**–**e**) were conducted across 10^3^ subsamples per sample size.

Figure 5a contrasts sample sizes on a human corpus callosum light microscopy section: a whole sample (~ 10^6^ axons) versus a subsample of 10^3^ axons, representative of typical human 2D microscopy datasets (e.g., refs. ^9,10^). The resulting distributions agree in the bulk, but differ in the tail, where the subsample shows occasional spikes rather than the smooth decay with increasing radius of the whole sample. Figure 5b shows the variation across repeated subsampling at sample size *n* = 10^3^ for both species. Variation is strongest for the large-axon tail in humans, reinforcing the impression from Fig. 5a that capturing the tail requires larger samples than the bulk.

To quantify these effects, Fig. 5c–e assess distribution shape and ensemble average radii as a function of sample size. The Wasserstein distance (see Fig. 5c) shows that at *n* = 10^2^, sampling errors can exceed the anatomical variation across ROIs (dashed lines), making it difficult to distinguish sampling noise from true anatomical differences. However, at *n* ≥ 10^3^, sampling error becomes much smaller.

For ensemble average radii (see Fig. 5d, e), $\bar{r}$ exhibits no bias and small variation (< 5%) across all sample sizes for both species, suggesting that sample size error is below the systematic bias from minor axis approximation in 2D (∣Bias∣ ≥ 10.5%, see Fig. 3d, e). In contrast, *r*_MRI_ shows substantial underestimation at small samples: human data exhibits approximately −30% bias at *n* = 10^2^, while rat data is less affected (~ −10%). The *r*_MRI_ error converges toward zero for larger sample sizes, faster for rats (~ 10^3^ to 10^4^ axons) than for humans (~ 10^5^ axons), underscoring that robust tail sampling requires adjustment to the species under study.

### Parametric distributions miss long tails

Beyond empirical distributions, parametric models offer a compact statistical representation of axon radius distributions. Given that previous attempts to determine the best parametric model^14^ used sample sizes (*n* < 10^4^) that may not capture long tails (see Fig. 5), we revisited parametric model identification at larger scale, using the 3D rat (pooled along and across axons) and 2D human data described above.

**Fig. 6:**
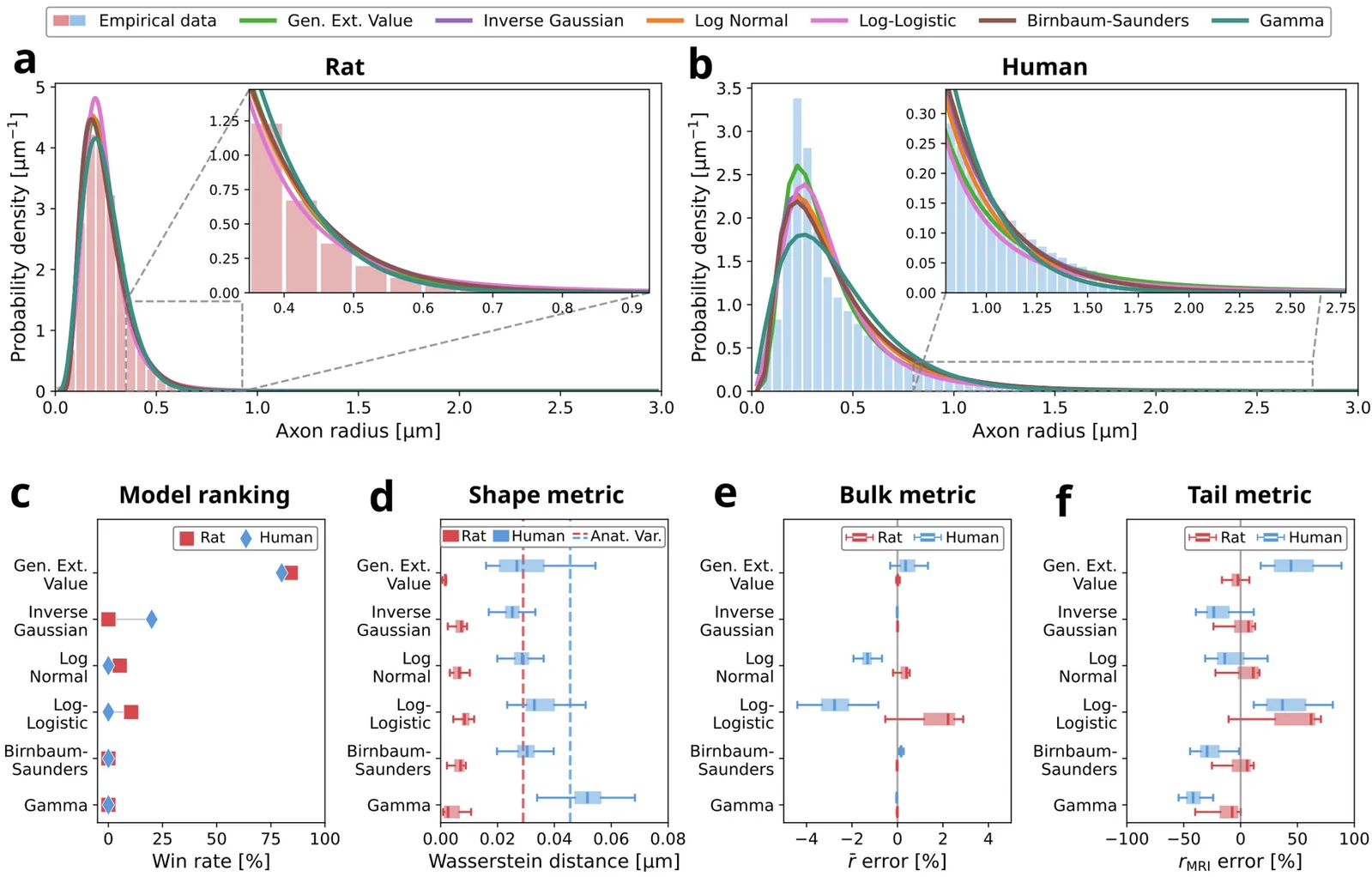
Parametric characterization of axon radius distributions. **a**, **b** Probability density pooled across ROIs for rat white matter and human corpus callosum with candidate parametric fits overlaid; insets emphasize the tail region. Fits are shown one per panel in Supplementary Fig. 4. **c** Candidate model ranking. The win rate quantifies the proportion of ROIs where each candidate distribution model ranks best in terms of Akaike information criterion (AIC). **d** Wasserstein distance (see Eq. (8)) between fitted and empirical distributions; vertical dashed lines indicate median inter-ROI anatomical variation for reference. **e**, **f** Relative error in ensemble average radii estimated from fitted distributions: **e** $\bar{r}$; **f** *r*_MRI_. Box markers in (**d**–**f**) summarize variation across ROIs: median (vertical line), inter-quartile range (IQR, box), and 1.5 × IQR (whiskers).

Figure 6a, b show pooled axon radius distributions for rat and human, with candidate parametric fits overlaid. For rat, the candidate distributions cluster tightly and fit the data well. For human, the fits diverge and most distributions fail to represent both the mode and the tail. Each fit is shown in a separate panel in Supplementary Fig. 4, providing a clearer view of these deviations.

To rank candidate models for individual ROIs, we used the Akaike information criterion (AIC), which balances goodness-of-fit against model complexity. Figure 6c shows the per-ROI win rate—the proportion of ROIs where each distribution achieves the lowest AIC. For both rat and human data, the generalized extreme value distribution wins in most ROIs, aligning with previous findings for long-tailed distributions in humans and monkeys ^14^. Figure 6d assesses whether goodness-of-fit translates to accurate estimation of distribution shape (Wasserstein distance). Results echo earlier findings: distributions cluster closely for rat, while human preserves the model ranking from (c). Overall, shape mismatch between parametric fits and empirical data falls well below anatomical variation for rats (dashed line); for humans, values approach anatomical variation, especially for the Gamma distribution, indicating poorer fit.

The ensemble average radii $\bar{r}$ and *r*_MRI_ (see Fig. 6e, f), reveal the mechanism behind the shape mismatch. While $\bar{r}$, sensitive to the bulk of the distribution, shows low error across parametric distributions and species (< 5%, Fig. 6e), *r*_MRI_, sensitive to the distribution tail, shows much larger errors (Fig. 6f). The effect is stronger in humans, where the heavier tail (see inset in Fig. 6b) leads to errors exceeding 25% for most distributions. Notably, the largest error occurs for the generalized extreme value distribution in humans—the best-ranked model by AIC—suggesting that goodness-of-fit criteria may not predict tail accuracy.

## Discussion

Using the largest available 3D and 2D white matter histology datasets, we examined along-axon radius variation and its functional implications, the robustness of axon radius distributions to histological sampling, and their parametric description. We found that, despite radius variation along individual axons, ensemble radius distributions remain stable along bundles, enabling 2D cross-sections to faithfully represent 3D bundle properties. Importantly, because along-axon variation has modest impact on conduction velocity, the representativeness of 2D cross-sections extends to conduction velocity predictions. In particular, large axons exhibit especially stable conduction, consistent with their key role in time-critical signaling. However, for diffusion MRI, along-axon variation has a larger impact, complicating interpretation. For histological sampling, we provide practical guidance: even small samples reasonably capture the distribution bulk, whereas larger samples are needed to characterize the long tail characteristic of human white matter. This long tail challenges parametric modeling of axon radius distributions, with standard parametric distributions failing to accurately capture tail-sensitive metrics.

Much of the field’s knowledge about axon organization derives from 2D histology, including differences across tracts^1,9,10,18^, species^12,17,33^ and health conditions^13,34^. While the consistency of 2D-based findings, e.g., in across-species differences, lends credibility to the approach, we provide explicit validation that 2D radius distributions robustly describe the local 3D distribution of radii. Thereby, we provide strong empirical validation of previous hints from approximately 50 manually traced monkey axons^22^—now across 19 fully segmented regions of interest (ROIs) totaling nearly 450,000 rat axons, including both traumatic brain injury (TBI) and control conditions.

The good capture of 3D axon radius distributions in 2D arises because radius variation is uncorrelated across axons and fluctuates only locally along axons, without systematic trends^35^. Consistent with this, 2D cross-section sampling in the data investigated here is statistically equivalent to random sampling from the pooled distribution across 2D cross-sections. The remaining systematic underestimation (~ 12%) arises from the minor axis approximation used in 2D, rather than from spatial structure within cross-sections. The circular equivalent approximation could provide less biased estimates (see Supplementary Fig. 3), but might require parametric filtering of obliquely cut axons for robust application.

Conduction velocity predictions often assume cylindrical axons^36,37^. Our results suggest this approximation holds in the presence of caliber variation: along-axon variation reduces conduction velocity by ~ 4% (median value) in the rat data investigated here, aligning with the small impact of focal axonal swellings in simulations^32^. To put this in perspective: estimated conduction velocity in human corpus callosum exceeds superficial white matter by ~ 20%^1^. CoV-related reductions would not overshadow this, even for extreme CoV differences—ranging from ~ 4% (CoV ≈ 0.27, the rat data investigated here) to ~ 12% (CoV ≈ 0.5^25^). The estimated conduction velocity reduction changes little when the constant myelin thickness assumed in our model is replaced by an empirically measured, radius-dependent one (see Supplementary Fig. 2). Practically, the robustness of both the cylinder model and 2D sampling to caliber variation reinforces findings from 2D histology-based conduction velocity research, ranging from tract-specific differences in conduction speed^1^ to cross-species scaling where larger brains harbor giant axons that partially offset longer conduction distances ^12^.

We find that large axons exhibit less relative variation (CoV ≈ 0.15) than smaller axons (up to CoV ≈ 0.30). While the rat axons in this study are smaller than those in primates, limited 3D monkey corpus callosum data (50 axons, ref. ^22^) confirms low variation (CoV ≈ 0.12) for large axons with mean radius ≈ 1.5 μm, suggesting this stability generalizes across species and larger axons in organized tracts. This likely reflects the special constraints on large axons: functionally, they serve as “highways” for time-critical activity^33^ and support high information rates that cannot be efficiently distributed across thinner axons^2^; metabolically, their costs scale with diameter squared^38^, favoring tighter optimization of these axons. This optimization of large axon conduction stability would follow a similar pattern established for g-ratio, where large axons cluster more tightly around the value that optimally balances conduction speed, energy efficiency, and volume constraints^1,25,37,39^.

Diffusion MRI offers a non-invasive window into axon microstructure by measuring water diffusion within axons. In this modality, the implications of along-axon radius variation are more nuanced than for conduction velocity. One class of approaches targets the axon radius distribution—either attempting to estimate a scalar proxy (*r*_MRI_)^7,40^ or the full distribution^5^. These approaches rely on the cylinder model, assuming axons have a characteristic radius distribution. Under this model, the radius distribution and *r*_MRI_ are in principle well-specified according to our findings and can be validated with 2D histology. This is also supported by recent experimental correlation between in vivo MRI- and histology-based *r*_MRI_ in the human corpus callosum^8^. That said, the diffusion MRI signal sensitive to axon radius coexists with diffusion MRI signal along axons in typical experimental regimes^7^. When falsely attributed to axon radius, this reduction in along-axon diffusion can reduce the specificity of radius estimates^28,29^.

Yet, radius variation along axons is not merely a confounder but contains measurable anatomical information. A recent study has shown that along-axon morphology can directly be probed with diffusion MRI, enabling discrimination between TBI and control rats^35^. There, the along-axon diffusivity is determined by the variance of cross-sectional fluctuations, i.e., by beadings and other caliber variations. Simulations in realistic axons show that these caliber variations, rather than undulations, dominate the time dependence of both diffusion and kurtosis along axons^28,29^. Cross-sectional variance along axons has further been proposed as one source of the microscopic kurtosis that correlation tensor MRI separates from other kurtosis sources^41,42^. Given that such approaches are sensitive to or even explicitly model deviations from the cylinder model, 3D histology is needed for validation.

While our findings suggest that 2D histological sampling of axon radius distributions is representative for 3D bundles, sample size remains a key consideration for designing histological studies. Our findings provide species- and application-dependent guidance for sample size selection, allowing researchers to balance sampling depth (within ROI) against width (e.g., across ROIs, donors, or conditions). Capturing the bulk of the distribution (e.g., $\bar{r}$) requires only modest sample sizes (~ 10^3^ axons), typically met in most published human histology studies (e.g., refs. ^9–13,43^). In contrast, capturing the tail is harder, requiring larger samples in humans (~ 10^5^ axons) due to their long distribution tail. Such sample sizes are only reached in recent large-scale 2D^1,8,44^ or 3D studies^25,26^. For practical application, sample sizes with accurate bulk capture may suffice for ensemble conduction velocity comparisons, while tail capture is needed for robust estimates of maximum conduction velocities or *r*_MRI_ reference data.

Parametric distributions offer a compact statistical description of axon radius distributions for informing biophysical models—from compound action potential simulations to brain network dynamics and diffusion MRI^5,37,45,46^. Aligning with previous findings^14,15^, our data confirms that the generalized extreme value distribution is a leading candidate across species according to the Akaike Information Criterion (AIC), but we show that model selection does not ensure accurate representation. While rat data is reasonably captured according to all evaluated metrics, human data with its long distribution tail shows poorer representation. This tail seems particularly hard to represent, as illustrated by large errors in the tail-weighted *r*_MRI_—paradoxically highest for the AIC-selected generalized extreme value distribution. Also of note, the Gamma distribution, despite its popularity in conduction velocity^16^ and diffusion MRI modeling^5^, also performs poorly. Overall, parametric modeling of axon radius distributions should be used with caution, particularly for applications that depend on accurate tail characterization.

Our study has several limitations. The 3D findings are derived from rat white matter, raising the question of whether they generalize to humans. However, monkey data—phylogenetically closer to humans—shows similar 2D–3D consistency and low along-axon variation for a limited dataset of 50 large axons (mean radius ≈ 1.5 μm, CoV  ≈ 0.12, reported by ref. ^22^), suggesting these properties hold for species with longer distribution tails. This is further supported by recent correlation between in vivo MRI- and histology-based *r*_MRI_—a tail-weighted metric—in the human corpus callosum^8^. We nonetheless suggest direct validation in large-scale human 3D axon reconstructions. Furthermore, we pooled ROIs from sham-operated and TBI rats, treating each as an individual observation rather than comparing across conditions. However, the 2D–3D agreement remains close to the identity line across all ROIs, suggesting that the results are not driven by pathology-related differences.

Our conduction model describes the axon as a sequence of local cylinders with circular cross-sections, combined through a slowness integral. The extracted radius profiles make the same approximation, so deviations from circular cross-sections are not captured (see Fig. 1a), but the description retains the strong local variation of the circular radius approximation caused by beadings and swellings (see Fig. 2a). In the model, only the distribution of local radii enters the slowness integral, not their order along the axon, whereas a saltatory model would depend on the spatial scale of the caliber variation relative to the internode length^47^. Furthermore, internodal length and node geometry are not represented, although both affect conduction velocity^48^. Their influence is nonetheless smaller than that of axon radius and g-ratio, which together account for about 85% of the variance in conduction velocity in a detailed axon model^49^. Finally, our model ignores axonal undulation (trajectory tortuosity). While Monte Carlo simulations suggest that caliber variation dominates over undulation for along-axon diffusion^28,29^, undulation may also affect conduction velocity, though the gentle undulations reported in rat white matter^35^ suggest a modest effect.

Our conclusions rest on observed radius distributions, which may differ from the true in vivo distributions. Tissue fixation causes shrinkage compared to in vivo conditions^9,50–53^, with recent work showing that shrinkage differs by axon radius^19^. Additionally, the human 2D light microscopy data cannot reliably resolve axons with radius below 0.3 μm ^44^.

Taken as a whole, our study reassesses a long-standing premise of white matter histology. Traditionally, researchers have used 2D histological sections to study axon radius distributions, implicitly assuming cylindrical axons. While 3D histology has falsified this assumption, we show that ensemble-level conclusions—including radius distributions and conduction velocity predictions—from 2D remain valid despite axon caliber variation. Beyond reinforcing decades of existing research, we provide guidance for efficient histological sampling across neuroscience applications.

## Methods

### Human corpus callosum (2D light microscopy)

We analyzed 2D histological sections from the human corpus callosum, comprising 46 million axons across 35 ROIs from two tissue samples (one male, 61 years; one female, 60 years; postmortem delay 20–24 h; see Fig. 1b, ref. ^8^). Case recruitment, acquisition of personal data, the protocols and the informed consent forms, performing the autopsy and handling the autopsy material were approved by the local ethics committees (Ethics Commission of the Medical Faculty of Leipzig University #205/17-ek; Ärztekammer Hamburg WF-74/16) in accordance with the Declaration of Helsinki (seventh revision, 2013) ^8^. Informed consent for the use of the tissue was obtained as part of this procedure. Whole brains were immersion-fixed in 3% paraformaldehyde and 1% glutaraldehyde in phosphate-buffered saline (pH 7.4). The corpora callosa were extracted and the ROIs were excised near the mid-sagittal plane. Tailored for MRI validation, each ROI spans approximately 3 × 3 mm—comparable to in-vivo MRI voxels—yielding roughly one million axons per ROI. The ROI blocks were contrasted with osmium tetroxide and uranyl acetate, dehydrated in graded acetones and embedded in Durcupan resin. Semi-thin sections (approximately 500 nm) were cut parallel to the mid-sagittal plane on a Reichert Ultracut II ultramicrotome, mounted on glass slides, stained with 1% toluidine blue and coverslipped. One light microscopy image per ROI was acquired with a Zeiss AxioScan Z1 slide scanner (40 × objective, numerical aperture 0.95, 0.11*μ*m/pixel). Myelinated axons were segmented with a U-Net ^54^ trained on manually annotated light microscopy subsections from separate donors, followed by connected-component labeling to identify individual axons ^44^; axons with radius below ~ 0.3 μm cannot be reliably detected at this resolution. Axon radii were estimated from the minor axis of ellipse fits to each segmented axon cross-section, which is more robust to obliquely cut axons than the circular equivalent radius; these radius measurements are provided in the public dataset^55^.

### Rat white matter (3D electron microscopy)

We used publicly available 3D electron microscopy data^24,56^, comprising segmented myelinated axons from the rat corpus callosum and cingulum (see Fig. 1a). The dataset includes serial block-face scanning electron microscopy volumes from 5 adult male Sprague-Dawley rats: 3 with traumatic brain injury (TBI) and 2 sham-operated controls, imaged 5 months post-surgery. All animal procedures in the original study were approved by the Animal Care and Use Committee of the Provincial Government of Southern Finland and performed according to the guidelines set by the European Community Council Directive 86/609/EEC ^24^. Both ipsilateral and contralateral hemispheres were sampled, yielding 10 volumes total. Each volume spans approximately 200 × 100 × 65 μm^3^ at 50 nm isotropic resolution, with approximately two-thirds corresponding to corpus callosum and one-third to cingulum, yielding 19 ROIs in total (10 volumes × 2 tracts, i.e., cingulum and corpus callosum, except for one volume without corpus callosum data; see Fig. 1a). The dataset provides automated segmentations of myelinated axons obtained with the DeepACSON pipeline, which combines deep convolutional neural network-based semantic segmentation with cylindrical shape decomposition for instance segmentation^24^. In total, approximately 450,000 myelinated axons are available.

#### ROI extraction

To separate each electron microscopy volume’s corpus callosum and cingulum populations (see Fig. 1a), we divided each volume into two spatial ROIs. For each ROI, we identified one of the image axes as the approximate bundle orientation, given that axons within each population are roughly aligned with coordinate axes.

#### Along-axon radius profiles

We extracted axon skeletons by tracing the medial axis via fast marching, using an implementation based on the original DeepACSON^24^ implementation. We sampled the perpendicular cross-section every 0.05 μm along the skeleton and computed the radius of a circle with equivalent segmented area.

#### 2D cross-sections

To mimic traditional 2D histology, we extracted 2D cross-sections along the image coordinate axis roughly aligned with the axon bundle orientation, accepting imperfect alignment as representative of experimental conditions. We estimated radii using the same minor-axis method as for the human data.

#### Outlier filtering

We manually inspected all axons with unusually large radii (*r*_MRI_ ≥ 1.5 μm, see Eq. (10)) and rejected non-axonal regions such as segmentation artifacts or blood vessels.

### Along-axon radius variation and its impact

#### Quantification of variation

From the rat 3D radius profiles, we quantified variation along individual axons using the coefficient of variation:

1 $${{\rm{CoV}}}=\dfrac{{\sigma }_{r}}{\bar{r}}$$

where *σ*_*r*_ is the standard deviation and $\bar{r}$ the mean radius along an axon’s trajectory. To ensure robust CoV estimates, we restricted analysis to axons exceeding 20 μm arc length, providing sufficient sampling along each axon’s trajectory.

#### Conduction velocity reduction

The conduction velocity of myelinated axons depends on the local radius *r* and g-ratio *g* as^36,37^

2 $$v(r,g)\propto r\sqrt{-\ln g}.$$

For an idealized cylindrical axon with $r=\bar{r}$ along the axon trajectory, this gives a uniform conduction velocity *v*_ideal_. For a realistic axon, however, variation of *r* and *g* along the axon trajectory leads to non-uniform velocity. The effective velocity over a segment of length *L* is

3 $${v}_{{{\rm{eff}}}}=\dfrac{L}{\int_{0}^{L}\dfrac{dx}{v(r(x),g(x))}}=\dfrac{1}{\langle {v}^{-1}\rangle },$$

and the conduction velocity reduction relative to the ideal cylinder is

4 $${{\mbox{Reduction}}}_{{{\rm{cond}}}}=1-\dfrac{{v}_{{{\rm{eff}}}}}{{v}_{{{\rm{ideal}}}}}.$$

We evaluated Eq. (4) from the sampled radius profiles along axon trajectories. To this end, we assumed constant myelin thickness *d*_*m*_ along the axon^22,57^ and a mean g-ratio of $\bar{g}=0.6$ near the conduction-optimal value^36,39^, so that the local g-ratio depends only on the local radius:

5 $$g(r)=\dfrac{r}{r+{d}_{m}},\qquad {d}_{m}=\bar{r}\,\dfrac{1-\bar{g}}{\bar{g}}=\dfrac{2\bar{r}}{3}.$$

Under these assumptions, Eq. (4) can be expressed in terms of CoV via Taylor expansion (see Supplementary Section S1):

6 $${{\mbox{Reduction}}}_{{{\rm{cond}}}}\approx \dfrac{c\,{{{\rm{CoV}}}}^{2}}{1+c\,{{{\rm{CoV}}}}^{2}},\qquad c\approx 0.53.$$

#### Along-axon diffusion reduction

For along-axon diffusion, caliber variations reduce the effective diffusivity compared to an ideal cylinder as^28^:

7 $${{\mbox{Reduction}}}_{{{\rm{diff}}}}\approx \dfrac{4\,{{{\rm{CoV}}}}^{2}}{1+4\,{{{\rm{CoV}}}}^{2}},$$

The factor of 4 relative to conduction velocity arises because diffusion is governed by cross-sectional area (*A* ∝ *r*^2^), so CoV^2^(*A*) = 4 CoV^2^(*r*).

### Axon radius distribution analyses

#### 2D-derived distributions as 3D proxies

To compare 2D and 3D representations of axon radius distributions, we extracted multiple 2D cross-sections perpendicular to the bundle from each 3D ROI. For each cross-section, we computed a 2D radius distribution (*P*_2D_) and compared it to the ROI’s 3D reference distribution (*P*_3D_), which pools radii measured along axon trajectories across all axons within the ROI. We quantified agreement using complementary metrics below.

To assess distribution shape, we used the Wasserstein distance (specifically the 1-Wasserstein or earth mover’s distance, ref. ^58^):

8 $$W({P}_{{{\rm{2D}}}},{P}_{{{\rm{3D}}}})=\int_{-\infty }^{\infty }| {F}_{{{\rm{2D}}}}(r)-{F}_{{{\rm{3D}}}}(r)| \,dr,$$

where *F*_2D_ and *F*_3D_ are the cumulative distribution functions corresponding to *P*_2D_ and *P*_3D_. As a reference for interpreting 2D–3D deviations, we computed the anatomical variation—defined as the median pairwise Wasserstein distance between 3D distributions from different ROIs.

To assess ensemble average radii, we compared two metrics: the arithmetic mean radius

9 $$\bar{r}=\langle r\rangle =\dfrac{1}{N}{\sum }_{i=1}^{N}{r}_{i},$$

which provides a bulk-weighted measure of typical axon size, and the MRI-visible axon radius^7,40^

10 $${r}_{{{\rm{MRI}}}}={\left(\dfrac{\langle {r}^{6}\rangle }{\langle {r}^{2}\rangle }\right)}^{1/4},$$

which weights larger axons more heavily and is particularly sensitive to the distribution tail.

To quantify systematic offset, we computed the normalized mean bias error

11 $${{\rm{NMBE}}}=\dfrac{1}{K}{\sum }_{k = 1}^{K}\dfrac{{m}_{{{\rm{2D}}},k}-{m}_{{{\rm{3D}}},k}}{{m}_{{{\rm{3D}}},k}},$$

where *m* denotes the metric of interest ($\bar{r}$ or *r*_MRI_), *k* indexes ROIs, and *K* is the number of ROIs.

To assess correlation between 2D and 3D estimates of $\bar{r}$ and *r*_MRI_, we mimicked a typical 2D histological study by sampling a single *P*_2D_ per ROI and computing Pearson’s *R* with the corresponding *P*_3D_ values across ROIs. We repeated this over 10,000 iterations and report *R* as mean ± standard deviation across iterations, with *p*-values derived from Monte Carlo permutation testing, estimating *p* as the fraction of permutations where the permuted *R* exceeded the observed *R*.

#### Per-cross-section versus random sampling

To test whether 2D–3D deviations arise from spatial structure within cross-sections, we repeated the above comparison with the single cross-section replaced by a random sample of matched size, drawn from the 2D radii pooled across all cross-sections of that ROI. We sampled with replacement, so that the random sample consists of independent and identically distributed radii, which is the null model the comparison tests.

#### Sample size requirements

To assess how sample size affects estimation accuracy, we performed a subsampling analysis using the 3D radius distributions from rat and 2D human radius distributions as population reference (both ~ 10^6^ or more radius samples per ROI). From each ROI’s full sample, we drew random subsamples of sizes *n* ∈ [10^2^, 10^5^] with 1000 repetitions per *n*. For each subsample, we quantified deviation from the full sample using *W* for distribution shape, and relative error in $\bar{r}$ and *r*_MRI_. This random subsampling assumes spatially uncorrelated axon radii, which is supported by our finding that 2D cross-section sampling is statistically equivalent to random sampling from the pooled distribution (see Fig. 4).

#### Parametric characterization

We evaluated six candidate parametric distributions, identified as top candidates by ref. ^14^: gamma, lognormal, generalized extreme value, inverse Gaussian, Birnbaum–Saunders, and log-logistic. For each distribution, we estimated parameters per ROI using binned maximum likelihood and evaluated goodness-of-fit using the Akaike Information Criterion (AIC). We quantified fit quality using *W* between fitted and empirical distributions, and bias in $\bar{r}$ and *r*_MRI_ by comparing values obtained via numerical integration of the fitted PDF, evaluated from 0*μ*m to the 99.999th percentile of pooled radii per species, to empirical values.

### Statistics and reproducibility

This study reanalyses two previously published, publicly available datasets; no new experiments were performed. Accordingly, the biological replicates were the subjects of the original studies—3 rats with traumatic brain injury, 2 sham-operated rats and 2 human donors—and the units of analysis were the white matter regions of interest (ROIs) segmented from them; no technical replicates were generated. The rat 3D dataset comprised approximately 450,000 myelinated axons across 19 ROIs from 10 imaged volumes (the ipsilateral and contralateral hemisphere of each animal); the human 2D dataset comprised approximately 46 million axons across 35 ROIs from the 2 donors. Along-axon statistics were restricted to axons with an arc length above 20 μm (*n* ≈ 150,000), and binned statistics required at least 50 axons per bin. Sex was recorded for both datasets but not analysed: the rat data comprise male animals only, and the human data comprise one male and one female donor, which is too few for a meaningful sex-based comparison.

Distribution shape was compared using the Wasserstein distance; the correlation between 2D- and 3D-derived radius estimates was quantified with Pearson’s *R*, together with the normalized mean bias; and parametric distribution fits were ranked by the Akaike Information Criterion. All *p*-values are one-sided and were obtained by Monte Carlo permutation testing with 10,000 permutations, using a fixed random seed to ensure reproducibility. All analyses were performed in Python 3.11 with NumPy 2.4.6, SciPy 1.17.1 and scikit-image 0.26.0; figures were generated with Matplotlib 3.11.1.

## Supplementary information

Transparent Peer Review file

Supplementary information

## Data Availability

The 3D electron microscopy rat data and its segmentation is publicly available at https://etsin.fairdata.fi/dataset/f8ccc23a-1f1a-4c98-86b7-b63652a809c3^24,56^. The axon radius distributions from 2D human light microscopy data are publicly available at https://zenodo.org/records/17431227^8,55^. The source data underlying all result figures are provided in the code repository (see Code availability).

## Code availability

All code to reproduce the results and figures from the datasets above is publicly available under the MIT license at https://doi.org/10.5281/zenodo.22286087. Figures can be regenerated either from the source data files contained in the repository or by reprocessing the public datasets from scratch.
