# Supplementary material for: White matter fiber bundles enable robust conduction and time-critical signaling despite axon caliber variation
Source: Commun Biol. 2026 Oct 27;9:1410. doi: 10.1038/s42003-026-11206-8 (PMC13714280; doi:10.1038/s42003-026-11206-8)
Supplement: Supplementary file 1 [file 42003_2026_11206_MOESM1_ESM.pdf]

nature portfolio

Peer Review File

White matter fiber bundles enable robust conduction and time-critical signaling despite axon caliber variation

Corresponding Author: Dr Laurin Mordhorst

This file contains all reviewer reports in order by version, followed by all author rebuttals in order by version.

Version 0:

Reviewer comments:

Reviewer #1

(Remarks to the Author)
This manuscript investigates the important question of how well 2D histology characterizes the 3D organization of axons in white matter fiber bundles. In particular, how well do the axon radius distributions from 2D cross-sections match those from 3D axon reconstructions. While much of our understanding of axon organization is based on 2D histology along with the assumption of cylindrical axons, recent 3D histology showing along-axon radius variation has raised questions about the validity of this approach. Using axon radius distributions extracted from a large 3D electron microscopy dataset of the rat corpus callosum and cingulum, this study addresses the critical need to validate the previous approach. The along-axon radius variation in the 3D dataset was quantified as the coefficient of variation (CoV). For this population, the CoV had a median of 0.27and showed a clear dependence on axon radius, with thicker axons showing less variation. The median estimated reduction in conduction velocity due to radius variation was small (~4%). The manuscript found that axon radius distributions obtained from cross-sections of the 3D dataset mimicking 2D histological sampling agreed well with those from the full 3D distribution, with a slight shift to smaller radii. It appears that this shift arises from the minor axis approximation used in 2D versus the perpendicular to centerline measurements in 3D, rather than a limitation of 2D sampling. Together these observations reinforce the validity of findings from 2D histology-based research on conduction velocity. In addition the manuscript investigates the sample size required to characterize axon radius distributions. Since these distributions are right-skewed, a considerably larger sample size is needed to capture the distribution's tail than the bulk of the distribution, a problem that increases in larger species. Similarly, parametric models fit the bulk distribution but struggle to fit the distribution tail. This is a solid, well-designed study that answers an important question. The results should be of considerable interest to researchers that model nerve conduction based on 2D histology.

I have a couple of recommendations:

1) The observation that 2D slice sampling is statistically equivalent to independent random sampling from the 3D distribution is interesting. The authors should consider moving supplemental figure S3 to the main text with some additional description.

2) In general the figures are good but many of the panels are too small and the details are hard to see. This is particularly true of figures: 3a (insets), 3b (shaded bands), 4b (shaded bands), 5a,b (colors of parametric fits are indistinguishable). The tan and light blue lines in figures 2 and 3 are particularly hard to see; the blue and red colors in figures 4 and 5 are much more visible. Additionally, the figure captions need to be checked to confirm that they match the figure (e.g. figure 4c. ... vertical dashed lines). Larger figures with more visible lines would improve the manuscript.

Reviewer #2

(Remarks to the Author)
The present work investigates whehther and how axonal caliber variation across the length of the axon may impact conduction velocity. The authors use 3D histology to map the caliber variation and use a simple model of conduction velocity to estimate its effect. The findings reveal that despite significant axon caliber variation along the long axis, the conduction velocity is only minimally impacted, and that the cylinder model is a good approximation. The authors also show that 2D histology captures the main metrics despite the variation observed in 3D.

This is a highly intriguing study that addresses a very important problem, its novelty is very high and it would certainly appeal to the readership of Comms Biol. I only have a few comments that the authors may want to consider:

1. Regarding the 2D vs. 3D comparisons, Figure 1 is quite confusing as it shows the 2D histology in 3D. It would be beneficial for readers like me to maybe more explicitly represent the differences in the acquisition strategies / quantification.

2. The model that was used for conduction is quite simplistic and assumes constant mylein thickness and g-ratio. I wonder whether that does not lead to a cicrular arguement vis-a-vis the correctenss of the cylindrical model? At least a discussion along those lines seems to be warranted.

3. To me it would seem that their findings would be of interest for methods such as correlation tensor MRI that aim to quantify the microscopic kurtosis - perhaps it would be worthwhile to comment how the findings here impact on this (and other) more advanced dMRI methods? E.g. discussing also those methods reflecting/detecting beading effects and undulations.

NOAM SHEMESH

Version 1:

Reviewer comments:

Reviewer #1

(Remarks to the Author)
I'm pleased with the revision of the manuscript; the authors have satisfied all my recommendations. The visibility of the figures is greatly improved. Moving supplemental figure S3 to the main text as figure 4 is done well and clarifies an important point. The new supplementary figure 4 is a good solution to the problem of not being able to distinguish the individual parametric fits.

Reviewer #2

(Remarks to the Author)
The auhtors have seriously and meticulously addressed all my comments. Congratulations on very impressive work! Noam Shemesh

Open Access This Peer Review File is licensed under a Creative Commons Attribution 4.0 International License, which permits use, sharing, adaptation, distribution and reproduction in any medium or format, as long as you give appropriate credit to the original author(s) and the source, provide a link to the Creative Commons license, and indicate if changes were made.
In cases where reviewers are anonymous, credit should be given to 'Anonymous Referee' and the source.
The images or other third party material in this Peer Review File are included in the article's Creative Commons license, unless indicated otherwise in a credit line to the material. If material is not included in the article's Creative Commons license and your intended use is not permitted by statutory regulation or exceeds the permitted use, you will need to obtain

permission directly from the copyright holder.
To view a copy of this license, visit https://creativecommons.org/licenses/by/4.0/

Authors’ Response to Reviews of

White matter fiber bundles enable robust conduction and time-critical signaling despite axon caliber variation

Laurin Mordhorst, Nikolaus Weiskopf, Markus Morawski, Siawoosh Mohammadi
Communications Biology

R: *Reviewers’ Comment*, A: Authors’ Response, RM: Revised Manuscript

General response

We thank both reviewers for their positive assessment and constructive comments. We have addressed all points and revised the manuscript accordingly.

Separately, we made a number of changes required by the editorial guidelines, listed under *Editorial requirements* at the end of this document. The main one is a shortened abstract.

Beyond the reviewers’ comments, we made a few corrections of our own, listed under *Author-initiated corrections* at the end of this document.

In the marked-up manuscript, added text appears in purple and ~~removed text in red, struck through~~. Line numbers quoted below (RM. Line n) refer to the marked-up manuscript.

R1. Reviewer #1

R1.C1. Comment 1

R: *The observation that 2D slice sampling is statistically equivalent to independent random sampling from the 3D distribution is interesting. The authors should consider moving supplemental figure S3 to the main text with some additional description.*

A: We moved the figure to the main text, where it now appears as Figure 4 in the Results section *2D cross-sections capture axon radius distributions*, directly after Figure 3, with its caption adapted to the style of the main manuscript figures.

We now describe the analysis in more detail in the Results section, rather than only referencing the figure (see RM. Line 126):

> ~~Figure S3 underlines this notion, showing that 2D slice sampling is statistically equivalent to independent random sampling from the 3D distribution.~~
>
> To test directly whether the residual 2D–3D differences reflect spatial structure within cross-sections, we compared actual cross-sections against random samples of matched size drawn from the 2D radii pooled across all cross-sections of each ROI. If axons of similar radius clustered within a cross-section, per-cross-section sampling would deviate more from the 3D reference than random sampling. Figure 4a–c repeat the comparisons of Figure 3c–e for both sampling schemes, covering distribution shape and the ensemble average radii $\bar{r}$ and r_{MRI}. Both schemes agree closely, with median Wasserstein distances of $0.028\,\mu\mathrm{m}$ (per cross-section) versus $0.027\,\mu\mathrm{m}$ (random), and near-identical correlations with the 3D reference for $\bar{r}$ ($R = 0.99 \pm 0.00$, $p < 0.001$ for both) and r_{MRI} ($R = 0.97 \pm 0.03$, $p < 0.001$ for both), suggesting that axon radius is spatially unstructured within cross-sections.

We slightly adjusted the Discussion, which previously referred to the supplementary figure (see RM. Line 212):

> The good capture of 3D axon radius distributions in 2D arises because radius variation is uncorrelated across axons and fluctuates only locally along axons, without systematic trends (Abdollahzadeh et al., 2025). ~~Fig. S3 implicitly shows that these conditions are met by demonstrating that~~ Consistent with this, 2D cross-section sampling in the data investigated here is statistically equivalent to random sampling from the pooled distribution across 2D cross-sections.

We added a Methods paragraph for the sampling procedure (see RM. Line 429):

> **Per-cross-section versus random sampling.** To test whether 2D–3D deviations arise from spatial structure within cross-sections, we repeated the above comparison with the single cross-section replaced by a random sample of matched size, drawn from the 2D radii pooled across all cross-sections of that ROI. We sampled with replacement, so that the random sample consists of independent and identically distributed radii, which is the null model the comparison tests.

R1.C2. Comment 2

R: *In general the figures are good but many of the panels are too small and the details are hard to see.*

A: We revised all result and supplementary figures to improve legibility. We increased font sizes throughout, in particular for the smallest labels, along with line widths and marker sizes.

R: *This is particularly true of figures: 3a (insets), 3b (shaded bands), 4b (shaded bands), 5a,b (colors of parametric fits are indistinguishable). The tan and light blue lines in figures 2 and 3 are particularly hard to see; the blue and red colors in figures 4 and 5 are much more visible.*

A: In Figure 3a, we enlarged the probability density plots containing the insets and increased the font size within the insets.

We made the shaded bands in Figures 3b and 5b (Figure 4b in the original) more visible. They now span the central $95\,\%$ of cross-sections and of subsamples, respectively, rather than the interquartile range. The bands were narrow because the underlying variation is genuinely small. In Figure 3b, we further removed the envelopes around the ensemble average radii, whose variability across cross-sections is already shown in Figure 3d–e.

For the parametric fits in Figure 6a–b (Figure 5a–b in the original), we found it hard to improve distinguishability by a color scheme or line styles, because the lines overlap strongly in many regions. In the main figure we therefore only increased the line width. Instead, we added Supplementary Figure 4, which shows each fit in a separate panel, following the presentation used by Sepehrband et al. (2016) for comparing parametric fits. We refer to it from the Results (see RM. Line 168) and from the caption of Figure 6:

> For human, the fits diverge and most distributions fail to represent both the mode and the tail. Each fit is shown in a separate panel in Supplementary Figure 4, providing a clearer view of these deviations.

We replaced the tan and light blue color coding by the red and blue that the reviewer found more visible. This affects Figures 2e, 3a–b, the new Figure 4a–c, and Supplementary Figure 3a.

R: *Additionally, the figure captions need to be checked to confirm that they match the figure (e.g. figure 4c. . . . vertical dashed lines).*

A: We checked all figure captions against their figures. In the caption of the sample size figure (Figure 5 in the revised manuscript, Figure 4 in the original), the dashed reference lines in panel (c) are horizontal, not vertical as stated:

> **(c)** Wasserstein distance as a function of sample size; ~~vertical~~horizontal dashed lines indicate median inter-ROI anatomical variation for reference.

We found and corrected a similar mismatch in the caption of Figure 6d.

The caption of Figure 4 (Supplementary Figure S3 in the original) stated that the random samples were drawn without replacement, whereas the analysis draws with replacement. We removed the statement from the caption and describe the sampling procedure in the Methods instead (RM. Line 429).

R: *Larger figures with more visible lines would improve the manuscript.*

A: We optimized the figures themselves within the current layout, as outlined above, and reduced the page margins so that all figures are now rendered larger. The final figure size is set by the journal's typesetting rather than by the template used here, and will likely exceed the size shown in this review version.

R2. Reviewer #2

R2.C1. Comment 1

R: *Regarding the 2D vs. 3D comparisons, Figure 1 is quite confusing as it shows the 2D histology in 3D. It would be beneficial for readers like me to maybe more explicitly represent the differences in the acquisition strategies / quantification.*

A: We agree that the segmentation panel did not sufficiently represent the three-dimensional nature of the data and could easily be mistaken for a 2D image. We redesigned Figure 1 accordingly.

Each panel now illustrates, for a single axon, how radius measurements are obtained in the two modalities. This emphasizes that 3D provides many measurements along one axon, whereas 2D provides a single measurement per axon, and it illustrates how the radius is approximated from the segmentation in each case: from a circle with the same area as the cross-section perpendicular to the centerline in 3D, and from the minor axis of a fitted ellipse in 2D.

While revising, we noticed that the Methods described the difference in quantification but did not motivate it. We now state why the minor axis is used in 2D, both in the caption of Figure 1 and in the Methods (see RM. Line 349):

> Axon radii were estimated from the minor axis of ellipse fits to each cross-section, which is more robust to obliquely cut axons than the circular equivalent radius; these radius measurements are provided in the public dataset (Mordhorst et al., 2025b).

The consequences of this choice are quantified in Supplementary Figure 3, which repeats the 2D–3D comparison using the circular equivalent radius instead.

R2.C2. Comment 2

R: *The model that was used for conduction is quite simplistic and assumes constant mylein thickness and g-ratio. I wonder whether that does not lead to a cicrular arguement vis-a-vis the correctenss of the cylindrical model? At least a discussion along those lines seems to be warranted.*

A: The g-ratio is not fixed in our model. We assume a constant myelin thickness d_m along the axon, following the 3D measurements along internodes of Andersson et al. (2020), and take a mean g-ratio from the literature only to calibrate d_m. The local g-ratio then follows the measured local radius, $g(r) = r/(r + d_m)$, and therefore varies along every axon.

To verify that our conclusions do not depend on this calibration, we repeated the analysis with an empirically measured, radius-dependent myelin thickness (Lee et al., 2019) in place of the constant one. The median reduction changes from $3.6\,\%$ to $4.3\,\%$, and the two curves converge for larger axons. We added this as Supplementary Figure 2 and state it in the Results (see RM. Line 96):

> Conduction velocity is much less affected than along-axon diffusion, with median reductions of $\sim$4 % and $\sim$21 % respectively. This also holds for an empirically measured, radius-dependent myelin thickness (see Supplementary Figure 2).

and in the Discussion (see RM. Line 226):

CoV-related reductions would not overshadow this, even for extreme CoV differences—ranging from $\sim$4% (CoV $\approx$ 0.27, the rat data investigated here) to $\sim$12% (CoV $\approx$ 0.5, Tian et al., 2025). The estimated conduction velocity changes little between the two myelin thickness models tested (see Supplementary Figure 2).

We agree that the cylinder model enters both the extracted radius profiles and the conduction model, and added a paragraph to the Limitations setting out what that model does not represent (see RM. Line 308):

Our conduction model describes the axon as a sequence of local cylinders with circular cross-sections, combined through a slowness integral. The extracted radius profiles make the same approximation, so deviations from circular cross-sections are not captured (see Fig. 1a), but the description retains the strong local variation of the circular radius approximation caused by beadings and swellings (see Fig. 2a). In the model, only the distribution of local radii enters the slowness integral, not their order along the axon, whereas a saltatory model would depend on the spatial scale of the caliber variation relative to the internode length (Scurfield and Latimer, 2018). Furthermore, internodal length and node geometry are not represented, although both affect conduction velocity (Arancibia-Cárcamo et al., 2017). Their influence is nonetheless smaller than that of axon radius and g-ratio, which together account for about 85 % of the variance in conduction velocity in a detailed axon model (Drakesmith et al., 2019). Finally, our model ignores~~We focused on along-axon radius variation but did not investigate~~ axonal undulation (trajectory tortuosity). While Monte Carlo simulations suggest that caliber variation dominates over undulation for along-axon diffusion (Lee et al., 2020, 2024), undulation may also affect conduction velocity, though the gentle undulations reported in rat white matter (Abdollahzadeh et al., 2025) suggest a modest effect.

We also made the claim itself more specific in the Discussion and Conclusion, bounding it to what we tested (see RM. Line 221, RM. Line 228, RM. Line 333):

Our results suggest ~~this is a reasonable approximation~~this approximation holds in the presence of caliber variation: along-axon variation reduces conduction velocity by $\sim$4% (median value) in the rat data investigated here, aligning with the small impact of focal axonal swellings in simulations (Kolaric et al., 2013).

. . . Practically, the ~~validity~~robustness of both the cylinder model and 2D sampling to caliber variation reinforces findings from 2D histology-based conduction velocity research . . .

. . . ensemble-level conclusions, including radius distributions and conduction velocity predictions, from 2D remain valid despite axon caliber variation.

R2.C3. Comment 3

R: *To me it would seem that their findings would be of interest for methods such as correlation tensor MRI that aim to quantify the microscopic kurtosis - perhaps it would be worthwhile to comment how the findings here impact on this (and other) more advanced dMRI methods? E.g. discussing also those methods reflecting/detecting beading effects and undulations.*

A: We extended the discussion of diffusion MRI accordingly (see RM. Line 260). We now name the along-axon features explicitly, add the kurtosis literature the reviewer refers to, and separate methods that target deviations from the cylinder model from those that are sensitive to them without modeling them:

Yet, radius variation along axons is not merely a confounder but contains measurable anatomical information. A recent study has shown that along-axon morphology can directly be probed with diffusion MRI, enabling discrimination between TBI and control rats (Abdollahzadeh et al., 2025). There, the along-axon diffusivity is determined by the variance of cross-sectional fluctuations, i.e., by beadings and other caliber variations. Simulations in realistic axons show that these caliber variations, rather than undulations, dominate the time dependence of both diffusion and kurtosis along axons (Lee et al., 2020, 2024). Cross-sectional variance along axons has further been proposed as one source of the microscopic kurtosis that correlation tensor MRI separates from other kurtosis sources (Alves et al., 2022; Henriques et al., 2021). Given that ~~this approach~~such approaches are sensitive to or even explicitly model~~s~~ deviations from the cylinder model, 3D histology is needed for validation.

Editorial requirements

We made the following changes to comply with the editorial requirements:

- We shortened the abstract to 159 words (RM. Line 7).
- We removed the two footnotes in the Methods and moved their content into the running text (RM. Line 365, RM. Line 383).
- We added a separate Methods section titled *Statistics and Reproducibility* (RM. Line 450).
- We cited the companion research articles alongside the datasets in the Data availability statement (RM. Line 470).
- We stated in the Methods that the human tissue and the animal procedures of the original studies were covered by the respective ethics approvals (RM. Line 344, RM. Line 360).
- We indicated in the abstract that the 3D reconstructions come from male rats, and state in the Methods why no sex-based analysis was performed, following the SAGER guidelines (RM. Line 7, RM. Line 460).
- We extended the competing interests statement to account for all authors (RM. Line 500).
- We added a correspondence line with the e-mail addresses of the two corresponding authors to the cover page.
- We made the code sharing compliant with the guidelines, replacing the repository link by a citable, persistent archive, which also contains the source data underlying the figures (RM. Line 477).
- We disclosed our use of large language models in the Acknowledgements, following the Nature Portfolio policy on artificial intelligence (RM. Line 485).

Author-initiated corrections

Beyond the reviewers' comments, we made the following corrections:

- We corrected the caption title of Supplementary Figure 3, which overstated the effect of the circular equivalent radius and did not match the corresponding section heading or the description in the Results:

  Circular equivalent radius ~~eliminates~~reduces bias but ~~reduces~~degrades precision.

- We now state in the Methods that Pearson's R is the mean $\pm$ standard deviation across Monte Carlo iterations, which the submitted manuscript reported without defining (RM. Line 427).
- We added a recent study on tissue shrinkage to the citations supporting the shrinkage limitation (RM. Line 326).
- We replaced "slice" by "cross-section" throughout the manuscript, for consistency with the term used elsewhere (RM. Line 216, RM. Line 378, RM. Line 440).
- We replaced "ground truth" by "reference" throughout, since the two terms were used interchangeably. The changes are in the captions of Figure 3, Figure 4 and Supplementary Figure 3, and at RM. Line 139 and RM. Line 436.
