# Supplementary material for: White matter fiber bundles enable robust conduction and time-critical signaling despite axon caliber variation
Source: Commun Biol. 2026 Oct 27;9:1410. doi: 10.1038/s42003-026-11206-8 (PMC13714280; doi:10.1038/s42003-026-11206-8)
Supplement: Supplementary file 2 [file 42003_2026_11206_MOESM2_ESM.pdf]

Supplementary Information for

White matter fiber bundles enable robust conduction and time-critical signaling despite axon caliber variation

Laurin Mordhorst[1,2*], Nikolaus Weiskopf[3,4,5],
Markus Morawski[3,6], Siawoosh Mohammadi[1,2,7*]

[1]Department of Neuroradiology, University of Luebeck, Luebeck, Germany.

[2]Institute of Systems Neuroscience, University Medical Center Hamburg-Eppendorf, Hamburg, Germany.

[3]Department of Neurophysics, Max Planck Institute for Human Cognitive and Brain Sciences, Leipzig, Germany.

[4]Felix Bloch Institute for Solid State Physics, Faculty of Physics and Earth System Sciences, Leipzig University, Leipzig, Germany.

[5]Functional Imaging Laboratory, Department of Imaging Neuroscience,
UCL Queen Square Institute of Neurology, University College London, London, UK.

[6]Paul Flechsig Institute - Centre of Brain Research and Neuropathology, Leipzig, Germany.

[7]Max Planck Research Group MRI Physics, Max Planck Institute for Human Development, Berlin, Germany.

[*]Correspondence: laurin.mordhorst@uni-luebeck.de, siawoosh.nathomohammadi@uni-luebeck.de

October 19, 2026

S1 Derivation of the conduction velocity reduction formula

Here, we derive Eq. (6) from the main text.

Velocity under constant myelin thickness. Substituting the g-ratio model from Eq. (5) into the velocity relation Eq. (2) gives

$$v(r) \propto r\left[-\ln g(r)\right]^{1/2} = r\left[\ln\left(1+\frac{d_m}{r}\right)\right]^{1/2}, \tag{S1}$$

where the velocity now depends only on the local radius r (since d_m is constant along the axon (Andersson et al., 2020; Behanova et al., 2022)).

Reduction in terms of slowness. Defining the slowness $h(r) = 1/v(r)$ (time per unit length), the transit time over a segment of length L is $t = L\,\langle h(r)\rangle$, so that $v_{\text{eff}} = L/t = 1/\langle h(r)\rangle$. Here, $\langle\cdot\rangle$ denotes the average over the local radii along an axon. The reduction from Eq. (4) then becomes

$$\text{Reduction}_{\text{cond}} = 1 - \frac{h(\bar{r})}{\langle h(r)\rangle}. \tag{S2}$$

where $\bar{r} = \langle r\rangle$ is the along-axon mean radius.

Taylor expansion. Writing $r = \bar{r}+\epsilon$ with $\langle\epsilon\rangle = 0$ and $\langle\epsilon^2\rangle = \text{CoV}^2\,\bar{r}^2$, a second-order Taylor expansion of h around $\bar{r}$ gives

$$\langle h(r)\rangle \approx h(\bar{r}) + h'(\bar{r})\underbrace{\langle\epsilon\rangle}_{=0} + \tfrac{1}{2}\,h''(\bar{r})\,\langle\epsilon^2\rangle = h(\bar{r}) + \tfrac{1}{2}\,h''(\bar{r})\,\text{CoV}^2\,\bar{r}^2. \tag{S3}$$

Substituting into Eq. (S2) gives

$$\begin{aligned}\text{Reduction}_{\text{cond}} &\approx 1 - \frac{h(\bar{r})}{h(\bar{r}) + \frac{1}{2}\,h''(\bar{r})\,\text{CoV}^2\,\bar{r}^2} \\ &= 1 - \frac{1}{1 + \underbrace{\frac{1}{2}\,\frac{h''(\bar{r})\,\bar{r}^2}{h(\bar{r})}}_{=c}\,\text{CoV}^2} \\ &= \frac{c\,\text{CoV}^2}{1 + c\,\text{CoV}^2}, \end{aligned} \tag{S4}$$

where c depends only on the dimensionless ratio $\varphi = d_m/\bar{r} = (1-\bar{g})/\bar{g}$, not on $\bar{r}$ itself. Computing $h''(\bar{r})$ from Eq. (S1) gives the closed-form expression

$$c(\varphi) = 1 - \frac{\varphi(3\varphi+4)}{4(1+\varphi)^2\ln(1+\varphi)} + \frac{3\varphi^2}{8(1+\varphi)^2\ln^2(1+\varphi)}. \tag{S5}$$

For $\bar{g} = 0.6$ (Chomiak and Hu, 2009; Rushton, 1951) (i.e., $\varphi = 2/3$), this yields $c \approx 0.53$.

S2 Absolute radius variation saturates for thick axons

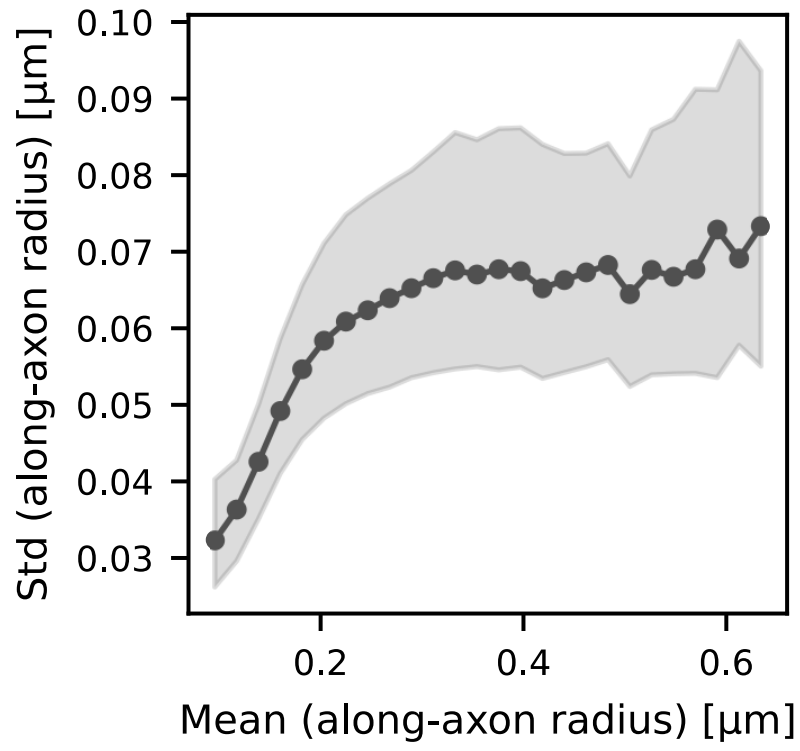

Supplementary Figure 1: **Absolute radius variation saturates for thick axons.** Standard deviation of radius along individual axons as a function of along-axon mean radius. Axons are binned by along-axon mean radius; line shows median, shaded region shows IQR. To robustly quantify along-axon radius variation, we computed statistics for axons exceeding 20 µm arc length ($n \approx 150{,}000$); bins were constrained to a minimum of 50 axons. The standard deviation saturates for thick axons, explaining why the coefficient of variation (CoV) in Fig. 2d decreases for thick axons.

S3 The conduction velocity reduction depends only weakly on the myelin thickness model

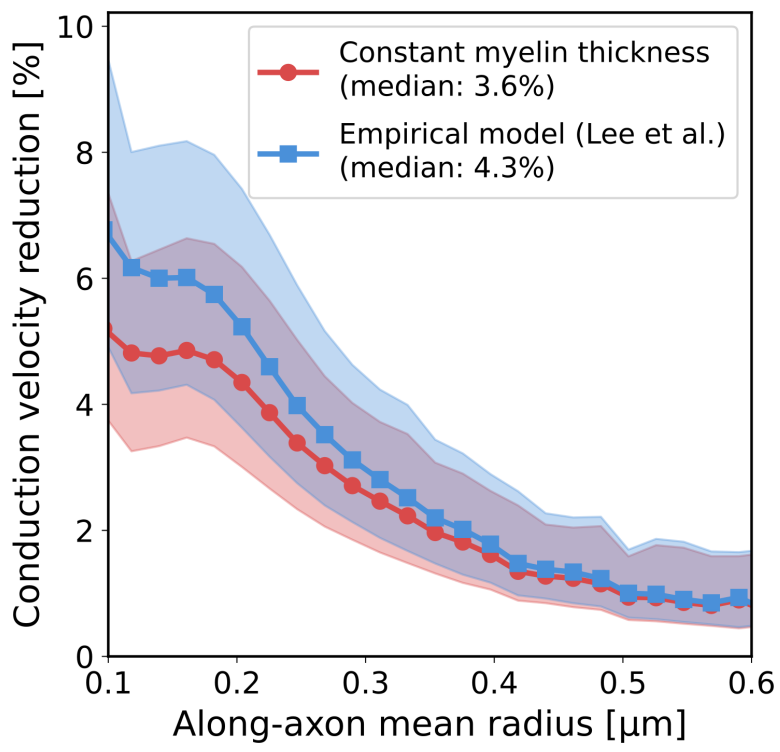

Supplementary Figure 2: **The conduction velocity reduction depends only weakly on the myelin thickness model.** Estimated reduction of conduction velocity (see Eq. (4)) as a function of along-axon mean radius, computed as in Fig. 2e. Red: constant myelin thickness d_m calibrated to a mean g-ratio of $\bar{g} = 0.6$ (see Eq. (5)), as used in the main analysis. Blue: myelin thickness measured in 3D electron microscopy by Lee et al. (2019), following the functional form of Berthold et al. (1983), $d_m(r) = 0.35 + 0.006\,(2r) + 0.024\,\ln(2r)$ in µm with r the local radius in µm; evaluated at each radius sample. Lines show medians across axons binned by along-axon mean radius, shaded bands show the interquartile range. The legend gives the median reduction across all axons.

S4 Circular equivalent radius reduces bias but degrades precision

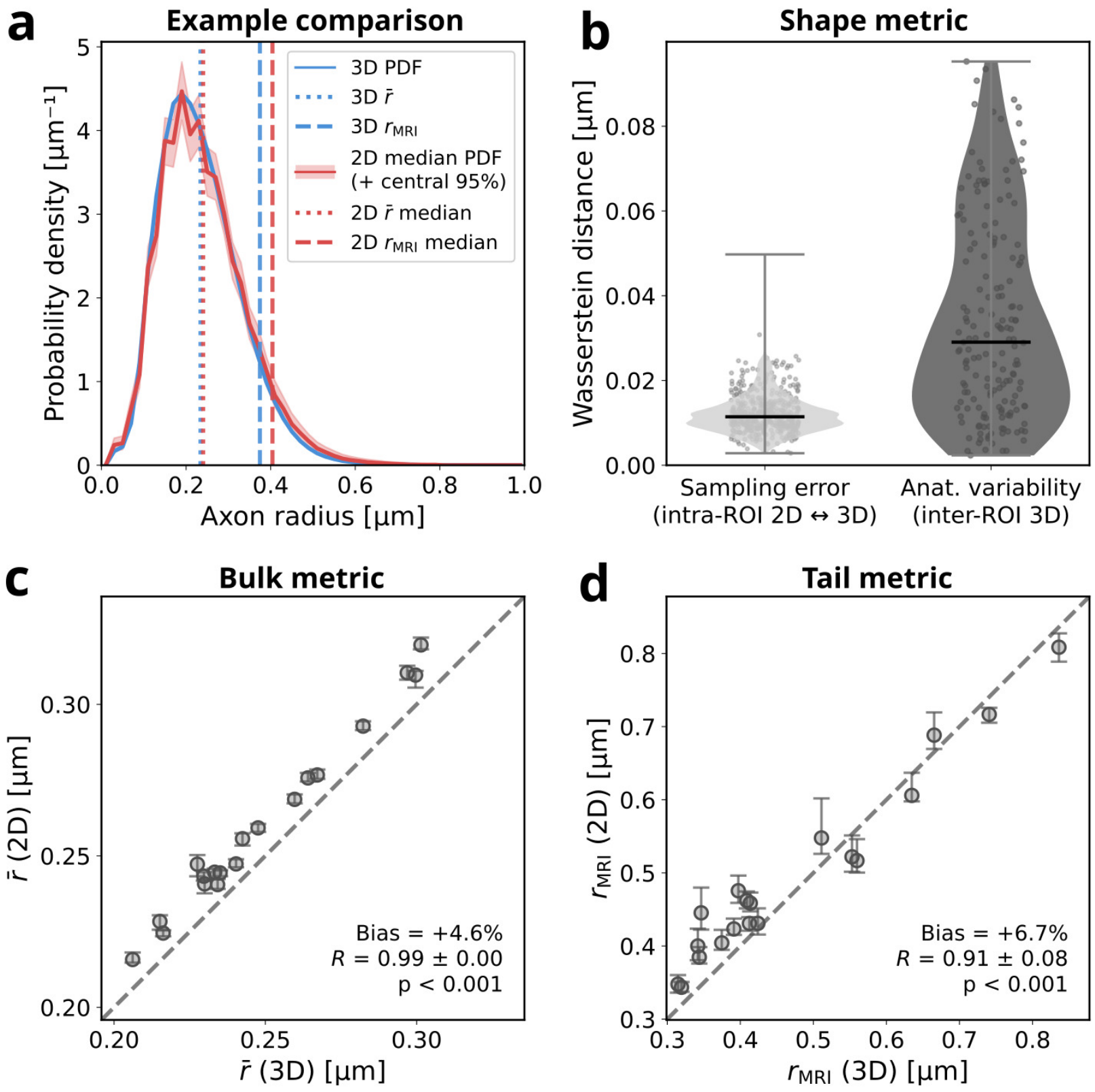

Supplementary Figure 3: **Circular equivalent radius reduces bias but degrades precision.** Same analysis as Fig. 3b–e, but using circular equivalent radius in 2D instead of the minor axis approximation. **(a)** Example comparison for one ROI. Solid lines show 2D and 3D-based radius distributions; vertical lines mark ensemble average radii: the arithmetic mean $\bar{r}$ (dotted, see Eq. (9)) is sensitive to the distribution bulk; the MRI-visible axon radius r_{MRI} (dashed, see Eq. (10)) is sensitive to the distribution tail. For 2D, lines indicate median values across cross-sections; the shaded band spans the central 95 % of cross-sections. **(b)** Distribution shape disagreement, quantified by Wasserstein distance (see Eq. (8)). The left distribution encodes all comparisons between distributions from 2D and their 3D counterpart, whereas the right distribution contextualizes this with inter-ROI distances between 3D distributions, reflecting the anatomical variation in the dataset. **(c–d)** Comparison of $\bar{r}$ and r_{MRI} from 2D cross-sections versus 3D reference across all ROIs. Markers show median and IQR across 2D cross-sections for each ROI; dashed lines indicate identity. Pearson's R and p-value were computed via Monte Carlo sampling.

S5 Parametric fits align with axon radius distributions in rats but less so in humans

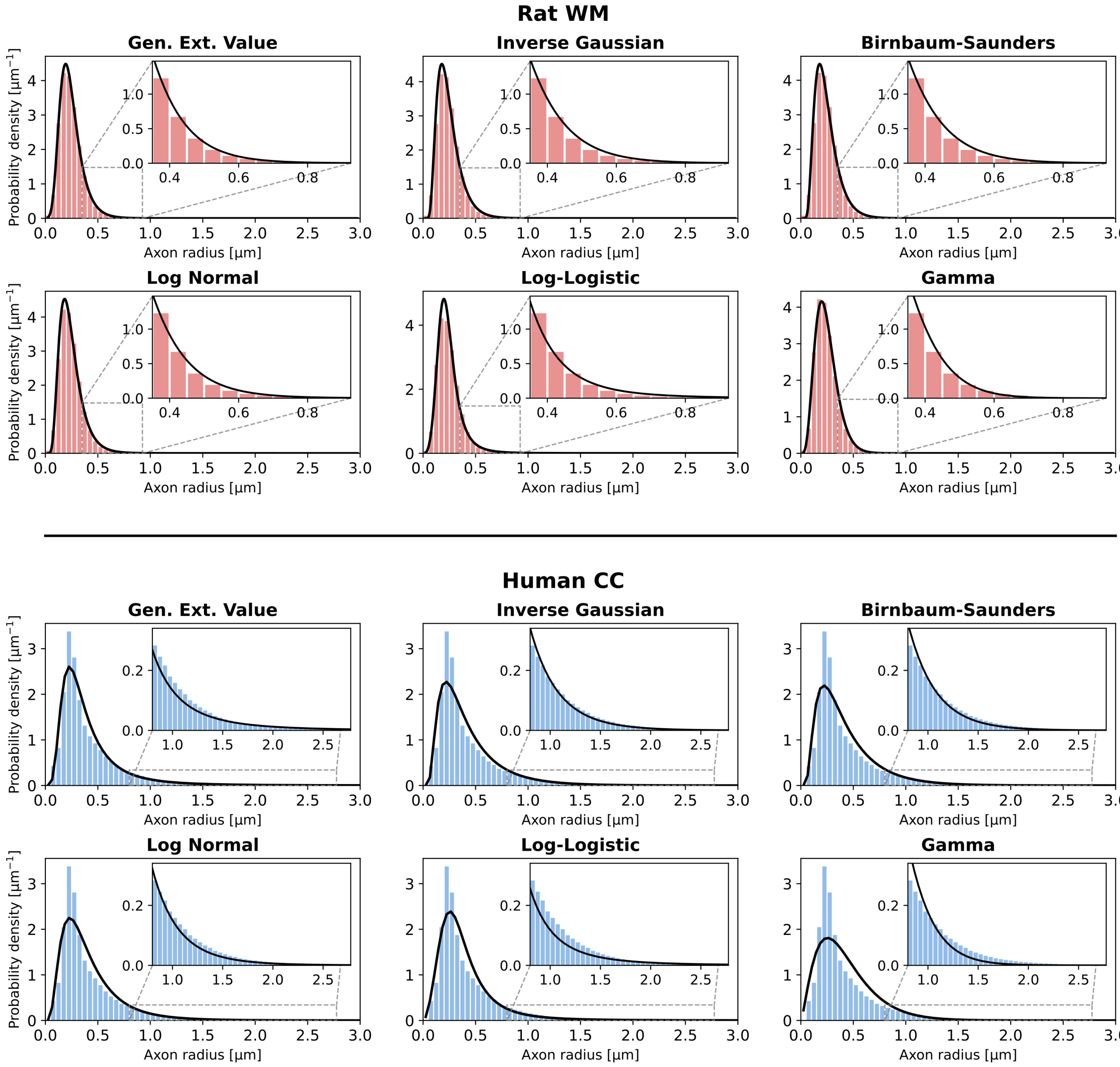

Supplementary Figure 4: **Parametric fits align with axon radius distributions in rats but less so in humans.** Parametric fits from Fig. 6a–b, shown separately for each candidate distribution in **(top)** rat white matter and **(bottom)** human corpus callosum. Each panel shows the probability density pooled across all ROIs of the respective dataset with the corresponding fitted distribution overlaid; insets emphasize the tail region.

References

Mariam Andersson, Hans Martin Kjer, Jonathan Rafael-Patino, Alexandra Pacureanu, Bente Pakkenberg, Jean-Philippe Thiran, Maurice Ptito, Martin Bech, Anders Bjorholm Dahl, Vedrana Andersen Dahl, and Tim B. Dyrby. Axon morphology is modulated by the local environment and impacts the noninvasive investigation of its structure–function relationship. *Proceedings of the National Academy of Sciences*, 117(52):33649–33659, December 2020. ISSN 0027-8424, 1091-6490. doi:10.1073/pnas.2012533117. URL http://www.pnas.org/lookup/doi/10.1073/pnas.2012533117.

Andrea Behanova, Ali Abdollahzadeh, Ilya Belevich, Eija Jokitalo, Alejandra Sierra, and Jussi Tohka. gACSON software for automated segmentation and morphology analyses of myelinated axons in 3D electron microscopy. *Computer Methods and Programs in Biomedicine*, 220:106802, June 2022. ISSN 0169-2607. doi:10.1016/j.cmpb.2022.106802. URL https://www.sciencedirect.com/science/article/pii/S0169260722001766.

C H Berthold, I Nilsson, and M Rydmark. Axon diameter and myelin sheath thickness in nerve fibres of the ventral spinal root of the seventh lumbar nerve of the adult and developing cat. *Journal of Anatomy*, 136(Pt 3):483–508, May 1983. ISSN 0021-8782. URL https://pmc.ncbi.nlm.nih.gov/articles/PMC1171896/.

Taylor Chomiak and Bin Hu. What is the optimal value of the g-ratio for myelinated fibers in the rat CNS? A theoretical approach. *PloS One*, 4(11):e7754, November 2009. ISSN 1932-6203. doi:10.1371/journal.pone.0007754.

Hong-Hsi Lee, Katarina Yaros, Jelle Veraart, Jasmine L. Pathan, Feng-Xia Liang, Sungheon G. Kim, Dmitry S. Novikov, and Els Fieremans. Along-axon diameter variation and axonal orientation dispersion revealed with 3D electron microscopy: implications for quantifying brain white matter microstructure with histology and diffusion MRI. *Brain Structure and Function*, 224(4):1469–1488, May 2019. ISSN 1863-2661. doi:10.1007/s00429-019-01844-6. URL https://doi.org/10.1007/s00429-019-01844-6.

W. a. H. Rushton. A theory of the effects of fibre size in medullated nerve. *The Journal of Physiology*, 115(1):101–122, 1951. ISSN 1469-7793. doi:10.1113/jphysiol.1951.sp004655. URL https://onlinelibrary.wiley.com/doi/abs/10.1113/jphysiol.1951.sp004655. _eprint: https://physoc.onlinelibrary.wiley.com/doi/pdf/10.1113/jphysiol.1951.sp004655.
